# Drivers of invertebrate community structure in a high-volume Neotropical cave system: a newly recognized subterranean biodiversity hotspot

**DOI:** 10.1007/s00442-026-05989-0

**Published:** 2026-10-16

**Authors:** Gabriel Augusto Silva Vaz, Rodrigo Lopes Ferreira, Marconi Souza-Silva

**Affiliations:** 1 Programa de Pós-graduação em Ecologia Aplicada, Universidade Federal de Lavras, Lavras, Minas Gerais CEP 37200-900 Brasil; 2 Centro de Estudos em Biologia Subterrânea, Departamento de Ecologia e Conservação, Instituto de Ciências Naturais, Universidade Federal de Lavras, Lavras, Minas Gerais CEP 37200-900 Brasil

**Keywords:** Caves, Troglobite, Non-troglobite, Large caves, Community ecology

## Abstract

Understanding how niche-based and neutral processes shape biological communities is a central objective in ecology, particularly in extreme and poorly explored environments such as large subterranean systems. Here, we investigated the mechanisms structuring terrestrial invertebrate communities in the one of the most extensive and voluminous cave systems in South America. Sampling across 56 sectors (30 m²) and 168 quadrats (1 m²) revealed 123 terrestrial invertebrate species. Complementary intuitive searches conducted throughout the cave, combined with standardized sampling, indicated that the system harbors at least 23 obligate cave species, supporting its recognition as a new hotspot of subterranean biodiversity. Total species richness, as well as the richness of non-troglobitic species, increased with resource availability and shelter availability, indicating a strong influence of niche partitioning. In contrast, troglobitic species richness declined markedly with increasing distance from aquatic environments, highlighting microclimatic stability as a key environmental filter. Beta diversity was primarily structured by microclimatic stability and resource availability, whereas geographic distance and cave identity had comparatively minor effects, suggesting that dispersal does not represent a major constraint on species distribution within this highly connected system. Resource gradients promoted species turnover in troglobitic assemblages, while richness differences predominated in non-troglobitic assemblages. Overall, our findings indicate that community assembly in large cave systems emerges from the joint action of environmental filtering and niche partitioning, with responses varying across spatial scales and degrees of ecological specialization. These results reinforce the conservation importance of high-volumnous cave systems as unique environments that harbor highly diverse and specialized biotas.

**Supplementary Information:**

The online version contains supplementary material available at https://doi.org/10.1007/s00442-026-05989-0.

## Introduction

One of the central aims of ecology is to identify patterns of biodiversity and to elucidate the mechanisms that generate and maintain them across spatial and temporal scales (Gaston 2000). This objective extends beyond the description of species distributions and community structure to encompass the investigation of the ecological and evolutionary processes underlying these patterns (Gaston 2000). At different spatial scales biodiversity is commonly structured by two overarching and non-exclusive sets of processes: neutral processes, such as dispersal limitation, and niche-based processes, including environmental filtering and niche partitioning (Nekola and White 1999; Vellend et al. 2014). Rather than operating independently, these processes frequently act in concert, jointly shaping the organization of biological communities (Gravel et al. 2006).

Neutral processes emphasize the role of stochasticity and dispersal constraints in community assembly. In particular, dispersal limitation assumes that species may be unable to reach or establish in environmentally suitable habitats despite favorable conditions for survival and reproduction, owing to intrinsic or extrinsic limits on dispersal capacity (Martín-Devasa et al. 2024). Therefore, increasing geographic separation among sites is expected to result in greater compositional dissimilarity among communities, reflecting reduced connectivity and limited colonization (Hubbell 2001; Nekola and White 1999). Such distance–decay relationships have been documented across both epigean and hypogean ecosystems, indicating that dispersal limitation represents a pervasive driver of β-diversity across contrasting environmental contexts (Mammola et al. 2019b).

In contrast, niche-based processes highlight the influence of species-specific ecological requirements, physiological tolerances, and biotic interactions on community assembly (Hubbell 2001). Environmental filtering refers to the selective exclusion of species unable to tolerate local abiotic conditions, such as climatic, microclimatic, or substrate-related constraints, thereby restricting community composition at the local scale (Mouillot, Demay and Tomasini 2007). Niche partitioning, in turn, emerges from differential resource use or microhabitat specialization, which reduces interspecific competition, facilitates species coexistence, and ultimately promotes higher levels of biodiversity within communities (MacArthur and Levins 1967; Souza-Silva et al. 2021).

Subterranean ecosystems provide a particularly informative framework for disentangling the relative contributions of neutral and niche-based processes to community assembly (Fiera et al. 2018; Kristjánsson et al. 2024). Although they remain among the least studied ecosystems worldwide (Mammola et al. 2021), caves are characterized by a set of distinctive and well-defined selective pressures in the dark zones, including the permanent absence of light, high environmental stability, and limited resource availability (Culver and Pipan 2013). Together, these conditions impose strong constraints on organismal persistence and performance (Cardoso et al. 2024; Mammola 2019). Notably, subterranean ecosystems frequently exhibit high β-diversity even across short spatial distances, further underscoring their relevance for studies of biodiversity patterns and processes (Mammola et al. 2020b).

Within subterranean systems, environmental filtering is widely recognized as a dominant driver of community assembly (Mammola et al. 2020a). The stringent abiotic conditions typical of caves restrict community membership to a relatively small subset of species capable of tolerating such environments, often reducing the relative importance of biotic interactions. Under these conditions, community composition is primarily determined by physiological tolerance rather than by competition or predation (Mammola and Isaia 2018; Prous et al. 2004). Consequently, cave communities are typically shaped by strong environmental filtering that favors highly specialized and evolutionarily distinct taxa (Bastos-Pereira et al. 2026; Mammola et al. 2024). Nevertheless, the relative influence of different ecological mechanisms may vary with spatial scale (Mammola et al. 2024). Even within a single cave, the mechanisms structuring communities can differ depending on the spatial resolution of analysis, indicating a strong scale-dependent effect (Junta et al. 2025). Moreover, the influence of these processes may also vary according to the degree of specialization to subterranean life, particularly when contrasting obligate cave species (troglobites) with non-troglobitic taxa (Mammola et al. 2019b).

Despite the growing body of research in subterranean ecology, the processes governing community assembly remain poorly understood in large, deep, and high-volume cave systems or integrated karst networks (e.g., Souza-Silva et al. 2021; Junta et al. 2025). Such systems may constitute especially valuable natural laboratories for investigating the interplay among ecological mechanisms, as they frequently harbor exceptionally high levels of invertebrate diversity (Nǎpǎruş-Aljančič et al. 2026). Many of these systems have been recognized as Hotspots of Subterranean Biodiversity (HSB), originally defined as caves hosting 20 or more troglobitic species (Culver and Sket 2000) and later revised to a threshold of 25 species (Culver and Pipan 2013).

In this context, the present study aims to identify the factors influencing species richness and β-diversity, as well as to evaluate the relative contributions of neutral and niche-based processes in shaping community assembly within a newly recognized subterranean biodiversity hotspot across multiple spatial scales (mesoscale and microscale). Specifically, we assess the roles of dispersal limitation, environmental filtering, and niche partitioning in structuring communities. These analyses were conducted for the entire invertebrate assemblage, as well as separately for troglobitic and non-troglobitic species. Additionally, we examine whether the caves composing the system share similar faunal composition, thereby testing the extent to which karst development influences functional connectivity among caves.

We hypothesize that microclimatic heterogeneity, acting through environmental filtering, constitutes the primary driver of terrestrial invertebrate richness and β-diversity. Furthermore, we predict that sites exhibiting greater microclimatic stability will support higher richness of troglobitic species, coupled with lower richness of non-troglobitic taxa.

## Materials and methods

### Study area

**Fig. 1:**
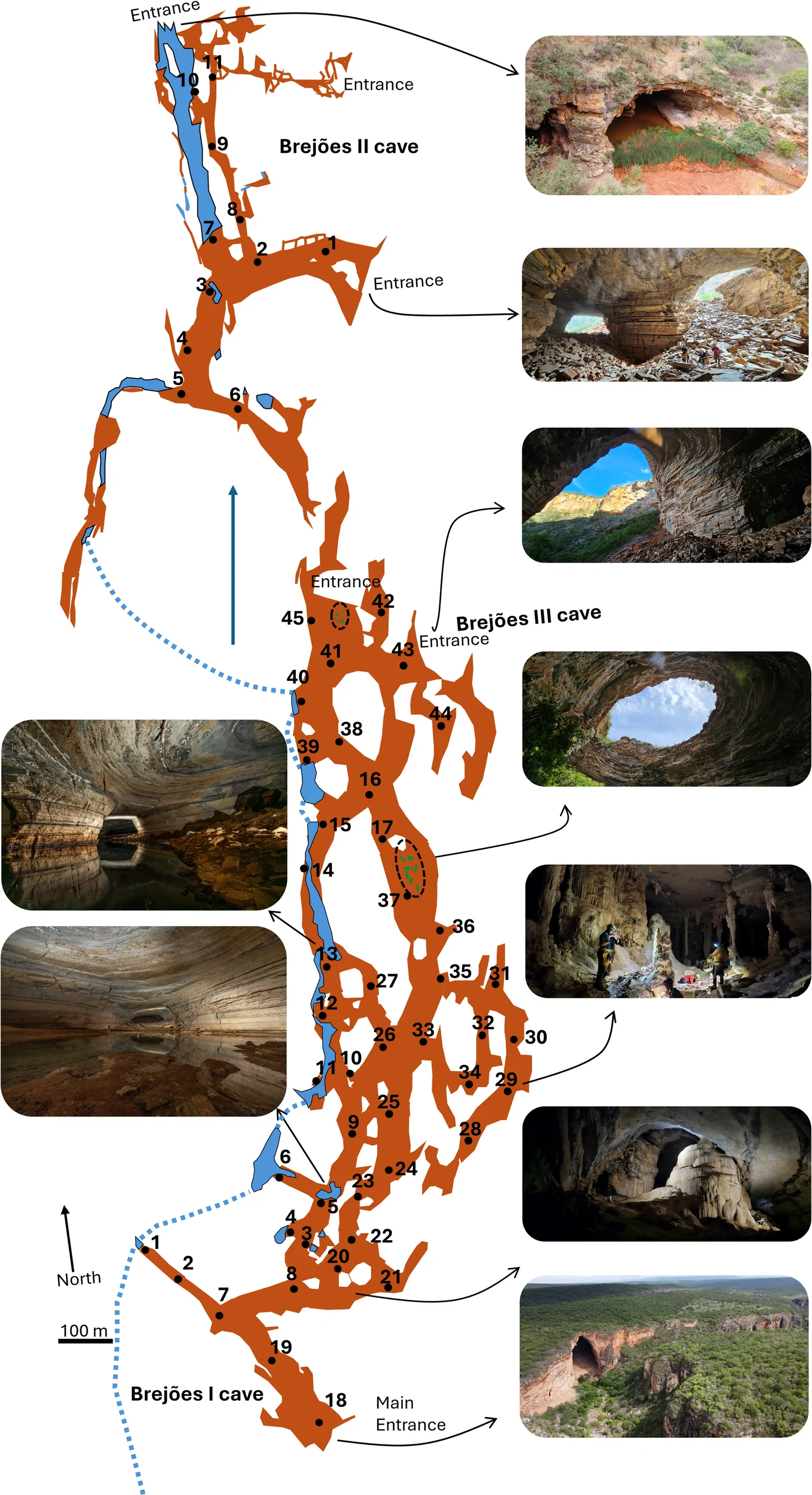
Brejões cave system map. Numbers and black dots represent the sampling units (sectors). The dashed blue line represents the cryptorheic portion of the Jacaré River. The blue arrow indicates the direction of the Jacaré River flow. Dashed circles with green dots represent vertical entrances (skylights). This map was adapted from the original maps of Brejões I and Brejões II, produced by the Bambuí Speleological Research Group and available in Rubbioli et al. (2019) and Furtado et al. (2022)

The study was conducted in the Brejões Cave System (-41.436021 S, -11.006843 W) located in the municipality of São Gabriel, Bahia State, northeastern Brazil (Fig. 1), within the Gruta dos Brejões/Veredas do Romão Gramacho Environmental Protection Area. The system is part of an extensive karst landscape developed in carbonate rocks of the Salitre Formation (Rubbioli et al. 2019).

The regional climate is classified as semi-arid (BSh) according to the Köppen–Geiger system, with mean annual precipitation below 700 mm (Alvares et al. 2013) and elevations ranging from 480 to 560 m a.s.l. (Berbert-Born and Karmann 2002). The dominant vegetation corresponds to seasonally dry tropical forest within the Caatinga domain, specifically the Irecê biogeographic district (Moro et al. 2024). This district is bordered by the Southern Sertaneja Depression and the Chapada Diamantina and is characterized by extensive limestone outcrops and a high density of caves. However, the region has experienced intense anthropogenic pressure, particularly deforestation driven by agricultural expansion (Moro et al. 2024).

The Brejões Cave System is composed of three main segments: Brejões I (upstream), Brejões II (downstream), and Brejões III. Brejões I and II are hydrologically connected by a sinking stream section of the Jacaré River, whereas Brejões III represents a lateral segment that is not connected to the river. (Berbert-Born and Karmann 2002; Furtado et al. 2022; Rubbioli et al. 2019; Fig. 1). Brejões I is the largest cave in the system, comprising approximately 8.4 km of mapped passages and Brejões II comprises approximately 2 km of mapped passages and exhibits a structural configuration broadly similar to that of Brejões I (Berbert-Born and Karmann 2002; Furtado et al. 2022; Rubbioli et al. 2019). More details about Brejões Cave System can be found in Supplementary material 1.

### Sampling design

Sampling followed a spatially structured design in which sectors, each measuring 10 × 3 m, were distributed along the cave passages of Brejões Caves I and II. Within each sector, three 1 m² quadrats were established (one positioned at the center and two at opposite ends) following the protocol proposed by Pacheco et al. (2020) and Souza-Silva et al. (2021). This hierarchical design enabled the collection of biological and environmental data at both the mesoscale (sector level) and microscale (quadrat level).

Across the Brejões Cave System, a total of 56 sectors were sampled, including 45 sectors in Brejões Cave I and 11 sectors in Brejões Cave II. Adjacent sectors were spaced at approximately 100 m intervals along the cave passages (Fig. 1). Sampling progressed from the main cave entrances toward deeper sections of the system, with biotic and abiotic variables systematically recorded within each sector and its associated quadrats.

Fieldwork was conducted over four consecutive days (19–22 January 2024), ensuring consistent sampling effort across spatial scales and cave segments, as well as during a resampling conducted in july 2024, when 24 of the 45 sectors in Brejões I were revisited (sectors: 1, 2, 3, 4, 5, 6, 9, 11, 12, 13, 14, 15, 16, 19, 20, 23, 26, 30, 34, 35, 39, 40, 41 and 43), along with their respective quadrats (*n* = 72). However, due to logistical constraints, not all sampling units could be resampled. Therefore, we used the second data in this study only for the description of the invertebrate fauna inventory.

### Characterizing and quantifying the cave floor microhabitat

Cave floor substrates were classified into three categories: trophic resources, shelter resources, and general substrates (i.e., substrates that do not provide shelter for invertebrates) (Rocha Melo et al. 2026) (see Table S1 and Fig. S2 A in Supplementary Material 1 for detailed descriptions of substrate classification and category assignment). In each sector, substrate characterization was conducted visually by the same observer, and quantification followed the protocol proposed by Pacheco et al. (2020) and Souza-Silva et al. (2021). This protocol consists of subdividing each sector along its longest axis into ten perpendicular sections. The percentage area occupied by each substrate component was estimated visually within these sections as proportions. Final values for each substrate type within a sector were obtained by averaging the proportional values across all sections.

At the microscale (quadrats), substrate characterization and quantification were performed using photographs taken in the field with a smartphone. These images were analyzed using ImageJ (Rasband 1997), which allowed the calculation of the surface area occupied by each substrate type (see Table S1 and Fig. S2B) based on pixel proportion.

Additionally, air temperature and relative humidity (microclimatic variables) were measured in each quadrat using a thermohygrometer (AKSO AK-625) positioned near the cave floor. Measurements were recorded only after the sensor readings had stabilized. For each sector, mean temperature and humidity values were calculated by averaging the measurements obtained from all quadrats within that sector.

### Distance measures

To quantify spatial relationships within the cave system, distances between each sector and cave entrances as well as aquatic environments were calculated by first plotting sector locations onto detailed cave maps. Distances were then measured in ImageJ following the actual orientation of cave conduits, rather than using straight-line (Euclidean) distances, thereby more accurately representing potential dispersal pathways within the subterranean environment. The distance from each sampling unit (i.e., sectors and quadrats) to the nearest cave entrance was used as a proxy for distance from the surface-deep zoneswithin an archipelago island biogeography framework (Rosindell and Phillimore 2011). In this context, the surface represents a larger and more stable source area, potentially harboring larger populations and lower extinction rates, and thus acting as a source of non-troglobite species to subterranean habitats. In contrast, deeper zones may be more isolated from the surface and may favor the persistence and local contribution of troglobite species. Additionally, the distance to the nearest aquatic environment was used as a proxy for microclimatic stability.

In addition, geographic coordinates for all sampling units were obtained by georeferencing the cave maps of the system (Rubbioli et al. 2019) in QGIS.

### Invertebrate sampling and identification

Invertebrates were surveyed using active visual search and manual collection techniques, following the methodology described by Souza-Silva et al. (2021). Specimens were collected with the aid of tweezers and fine brushes. Sampling followed a concurrent–sequential approach, beginning at the microscale with the quadrats, which were surveyed individually by one collector each, and subsequently expanding to the corresponding sectors, which were sampled simultaneously by three collectors.

To ensure comprehensive coverage within each sampling unit, all collectors actively participated in the search, and sampling continued until all visible invertebrates were collected or reliably accounted for. Search duration varied among sampling units, reflecting structural heterogeneity within the cave, such as differences in substrate complexity, the presence of rocks, crevices, and ledges (Sharratt et al. 2000; Souza-Silva et al. 2021).

In addition to the standardized sampling protocol, supplementary exploratory searches were conducted throughout the cave using direct and intuitive collection methods. These non systematic surveys were designed specifically to improve the detection of cave-restricted taxa by targeting a wide range of microhabitats that may not be fully represented within the predefined sampling units (Wynne et al. 2019). Specimens collected during these supplementary searches, conducted outside the established sectors and quadrats, were not incorporated into the quantitative ecological analyses presented in this study. Instead, these records were used exclusively to compile a comprehensive inventory of the cave fauna, with particular emphasis on identifying troglobitic species.

Collected specimens were immediately preserved in vials containing 70% ethanol. Subsequently, samples were sorted under a stereomicroscope at the Center for Studies in Subterranean Biology (CEBS), Federal University of Lavras (UFLA). Specimens were identified to the lowest possible taxonomic level using appropriate identification keys and were grouped into morphotypes following the approach proposed by Oliver and Beattie (1996).

Potential troglobitic species were identified based on the presence of troglomorphic traits (reduced or absent eyes and pigmentation, as well as elongated sensory and/or locomotory appendages) which are generally interpreted as indicators of long-term isolation and evolutionary adaptation to subterranean environments (Sket 2008) (see material supplementary 1 for more details). Finally, all specimens were deposited in the Collection of Subterranean Invertebrates of Lavras (ISLA) housed at the Center for Studies in Subterranean Biology, Federal University of Lavras (www.biologiasubterranea.com.br).

## Data analysis

### Exploratory analysis

Prior to model fitting, we evaluated collinearity among both predictor and response variables following the protocol recommended by Zuur et al. (2009). Response variables included total terrestrial invertebrate richness (S), non-troglobitic terrestrial invertebrate richness (St), and troglobitic terrestrial invertebrate richness (Snt). Predictor variables exhibiting Pearson correlation coefficients greater than 0.7 were considered redundant, and in such cases only one variable was retained for subsequent analyses.

Associations among continuous and categorical variables were initially explored graphically using boxplots and pairwise plots generated with the ggpairs function from the GGally package (Schloerke et al. 2025). This exploratory analysis revealed six pairs of predictors with potential collinearity: habitat type and temperature; habitat type and humidity; habitat type and distance to the nearest aquatic environment; resource presence and resource availability (quantity); resource presence and temperature; and resource presence and humidity. In addition, three pairs of variables were identified as clearly redundant: general substrate and shelter (sectors: *r* = − 0.94; quadrats: *r* = − 0.86), substrate diversity and shelter diversity (sectors: *r* = 0.83; quadrats: *r* = 0.72), and total richness (S) and non-troglobitic richness (Snt) (sectors: *r* = 0.98; quadrats: *r* = 0.97) (Figs. S3, S4). Based on these results, we excluded habitat type (i.e., margins of aquatic environments vs. other cave areas), shelter diversity, general substrate, and non-troglobitic richness from the subsequent analyses.

We also tested how habitat (margins of aquatic environments vs. the remaining regions of the Brejões Cave System) affects species richness and environmental variables (see Supplementary Material 1 for further details). These analyses were used to define the variables included in our models in the subsequent sections.

Based on these results, we excluded habitat type (i.e., margins of aquatic environments vs. other cave areas), shelter diversity, general substrate, and non-troglobitic richness from the subsequent analyses. All analyses described here and in the following sections were performed using R software, version 4.4.0 (R Development Core Team 2024).

### Richness analyses

Based on the results of the exploratory analyses, a reduced set of predictors was selected to minimize multicollinearity while retaining variables of clear ecological relevance. The resulting full model structure (in R notation) was specified as:

$$\begin{aligned}&Y\;\sim\;temperature\;+\;humidity\;\\&+\;shelter\;+\;substrate\;diversity\;\\&+\;resource\;availability\;+\;resource\;presence\\&\;+\;dis\tan ce\;to\;nearest\;aquatic\;environment\\&\;+\;dis\tan ce\;to\;nearest\;entrance\end{aligned}$$

Although resource presence exhibited some visual association with other predictors (Figs. S3, S4), it was retained in the initial model set due to its biological relevance and because it did not substantially inflate model variance.

We used generalized linear mixed models (GLMMs), implemented in the glmmTMB package (Brooks et al. 2017), to evaluate the factors influencing species’ richness. All continuous predictor variables were standardized (subtracting the mean and dividing by the standard deviation) to improve model convergence and facilitate direct comparison of effect sizes (Zuur, Ieno and Elphick 2010). The mixed-effects structure accounted for pseudoreplication, associated with the spatial hierarchy of sectors and quadrats.

We reassessed multicollinearity using variance inflation factors (VIFs) calculated with the check_collinearity function from the *performance* package (Lüdecke et al. 2021). Unlike the Pearson correlation coefficient used in the exploratory analysis, VIF evaluates multicollinearity within the GLMM model structure. The results confirmed that our full models did not present problematic multicollinearity (all models exhibited low collinearity (VIF < 5). Model assumptions, including residual distribution and potential zero inflation, were evaluated using the simulateResiduals and testZeroInflation functions from the *DHARMa* package (Hartig 2022).

To address any remaining redundancy and identify the most parsimonious models, we subsequently applied a model selection procedure followed the protocol of Burnham and Anderson (2002), using the *MuMIn* package (Bartoń 2023). Candidate models were generated and ranked using the dredge function, and models with ΔAIC < 2 were considered equally supported. Final parameter estimates and marginal and conditional R² values were obtained by calculating Akaike-weighted averages across the selected models using the avg.models and r.squaredGLMM functions.

### Beta diversity analysis

Species compositional dissimilarity (β-diversity) between pairs of sampling units was quantified at both the sector and quadrat scales using the beta function from the *BAT* package (Cardoso et al. 2015). We calculated abundance-based Sørensen dissimilarity (argument abund = TRUE). Total β-diversity was subsequently partitioned into two additive components: species replacement between sampling units (β-replacement) and differences in species richness between them (β-richness) (Cardoso et al. 2015).

Variation in species dissimilarity among sectors and among quadrats was then modeled using generalized dissimilarity modeling (GDM) (Ferrier et al. 2007), implemented in the *gdm* package (Fitzpatrick et al. 2024). This framework allows for nonlinear relationships between compositional dissimilarity and environmental gradients, as well as geographic distances among sampling units (Mokany et al. 2022). Model outputs and uncertainty in the fitted I-splines were visualized using *ggplot2* (Wickham 2016), based on results from the plotUncertainty function, which estimates uncertainty via bootstrapping.

GDM analyses were performed for total β-diversity, β-replacement, and β-richness using all terrestrial invertebrate species at both the sector and quadrat scales. In addition, separate analyses were conducted for troglobitic and non-troglobitic species, but only at the mesoscale (sectors), where sampling captured a sufficiently large proportion of the terrestrial troglobite assemblage to allow robust modeling.

Predictor variables included those retained following the pre-analysis collinearity screening (see model structure in richness analysis section). In addition, we incorporated cave identity (two levels: Brejões I and Brejões II) to explicitly test the effect of connectivity between caves within the system, as well as geographic distance between sampling units, which was included to account for spatial structure and dispersal-related processes.

Finally, Table 1 summarizes the relationships between the retained predictors and the ecological processes and mechanisms they are assumed to represent.

**Table 1:** Shows the relationship between the variables used in the models and the underlying ecological processes and mechanisms

Predictor variables	Ecological processes	Mechanisms
Distance to the near entrance (m)	Neutral	Dispersion
Geographic Distance	Neutral	Dispersion
Distance of aquatic environment (m)	Niche	Environmental filtering
Temperature (°C)	Niche	Environmental filtering
Humidity (%)	Niche	Environmental filtering
Resource (%)	Niche	Environmental filtering and niche partitioning
Substrate diversity (H’)	Niche	Niche partitioning
Shelter (%)	Niche	Niche partitioning

## Results

### Invertebrate fauna

The Brejões Cave System supports an exceptionally rich invertebrate fauna. Considering all sampling approaches, including standardized surveys in sectors, quadrats, and complementary searches in additional habitats, as well as a resampling conducted in July 2024, a total of 146 invertebrate species were recorded. These taxa span at least 39 orders and 80 families, with Araneae being the most species-rich group (34 species), followed by “Acari” (21 species), Diptera (16 species), and Coleoptera (11 species) (Supplementary Material 2).

**Fig. 2:**
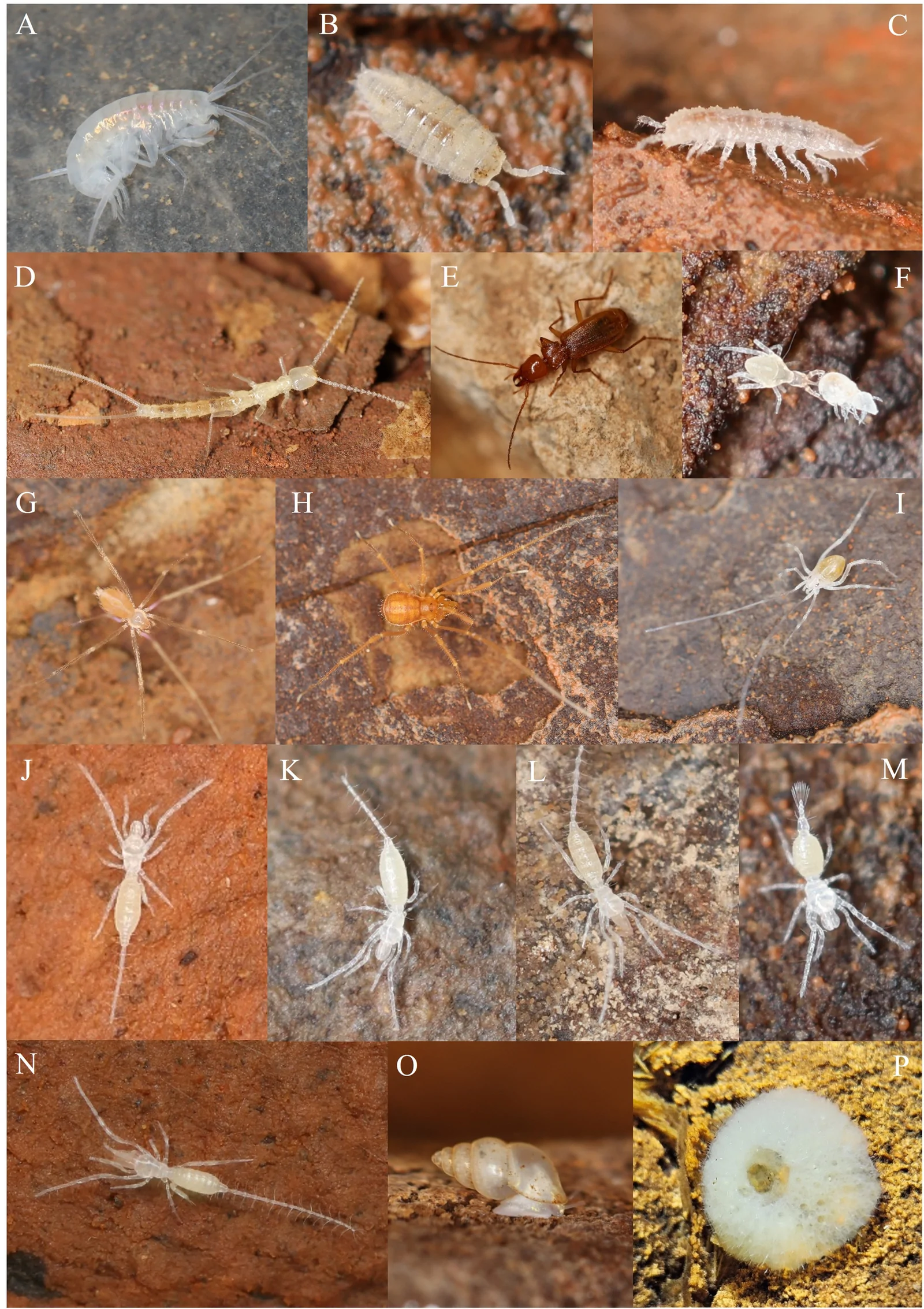
Some troglobitic species from the Brejões System. **A*** Spelaeogammarus quilombola* (Amphipoda: Artesiidae) Galo, Cardoso and Ferreira 2025. **B*** Trichorhina* sp. (Isopoda: Platyarthridae). **C*** Pectenoniscus morrensis* (Isopoda: Stylonicidae) Cardoso et al. 2020. **D** Projapygidae sp. (Diplura). **E*** Coarazuphium* sp. (Coleoptera: Carabidae). **F** Cunaxidae sp. (“Acari”:Trombidiformes) and Neelidae sp. (Collembola: Neelipleona). **G*** Ochyocera* sp. (Araneae: Ochyroceratidae). **H** Escadabiidae sp. (Opiliones: Laniatores). **I*** Neocarus* sp. (Opilioacarida: Opilioacaridae). **J***Eukoenenia* sp.1 (Palpigradi: Eukoeneniidae) **K***Eukoenenia* sp.2. **L***Eukoenenia* sp.3. **M***Allokoenenia* sp. (Palpigradi: Eukoeneniidae). **N***Eukoenenia* sp.4. **O** Cochliopidae sp. (Gastropoda: Littorinimorpha). **P** *Racekiela cavernicola* (Porifera: Spongillidae) Volkmer-Ribeiro, Bichuette & Machado 2010

Remarkably, the system harbors at least 23 troglobitic species, of which only three have been formally described. (Table 2 and Fig. 2). Based on the richness of obligate subterranean taxa, we propose that the Brejões Cave System be recognized as a new hotspot of subterranean biodiversity in South America.

**Table 2:** Taxonomic diversity and distribution of 23 troglobite species and morphotypes within Brejões Cave System. The study includes observations from two specific caves Brejões I Cave (BI), Brejões II Cave (BII). Additionally, the study distinguishes fauna between terrestrial (T) and aquatic (A)

Class	Order or taxa	Family	Species and Morphotypes	BI	BII	Habitat
Archnida	“Acari”	unidentified	Acari sp. 14	+		T
		Cunaxidae	Cunaxidae sp.	+		T
		Opilioacaridae	*Neocarus* sp.	+	+	T
		Rhagidiidae	Rhagidiidae	+		T
	Araneae	Ochyroceratidae	*Ochyocera* sp.	+	+	T
	Opiliones	Escadabiidae	Escadabiidae sp.	+		T
	Palpigradi	Eukoeneniidae	*Allokoenenia* sp.	+	+	T
		Eukoeneniidae	*Eukoenenia* sp. 1	+		T
		Eukoeneniidae	*Eukoenenia* sp. 2	+		T
		Eukoeneniidae	*Eukoenenia* sp. 3	+		T
		Eukoeneniidae	*Eukoenenia* sp. 4	+		T
	Pseudoscorpiones	Chtoniidae	*Pseudochtonius* sp.	+		T
Collembola	Entomobryomorpha	unidentified	Entomobryomorpha sp. 2	+		T
	Neelipleona	Neelidae	Neelidae sp.	+	+	T
	Symphypleona	Arrhopalitidae	Arrhopalitidae sp.	+		T
Desmospongia	Spongillida	Spongillidae	*Racekiela cavernicola* Volkmer-Ribeiro, Bichuette & Machado 2010	+		A
Diplura	Diplura	Projapygidae	Projapygidae sp.	+		T
Gastropoda	Stylommatophora	Streptaxidae	*Streptartemon* sp.	+		T
	Littorinimorpha	Cochliopidae	Cochliopidae sp.	+		A
Insecta	Coleoptera	Carabidae	*Coarazuphium* sp.	+		T
Malacostraca	Amphipoda	Artesiidae	*Spelaeogammarus quilombola* Galo, Cardoso & Ferreira 2025	+		A
	Isopoda	Plathyarthridae	*Trichorhina* sp.	+		T
		Stylonicidae	*Pectenoniscus morrensis* Cardoso et al. (2020)	+		T

Focusing exclusively on sampling using sectors and quadrats conducted in January 2024, we recorded 123 terrestrial invertebrate species across the 56 sampled sectors (mean richness = 8.02 ± 7.48 SD) and 67 species across the 168 sampled quadrats (mean richness = 1.64 ± 1.87 SD). Among these, thirteen troglobitic species were recorded at the sector level (mean = 0.78 ± 1.14 SD) and eight at the quadrat level (mean = 0.16 ± 0.44 SD). In contrast, non-troglobitic taxa accounted for 110 species in sectors (mean = 7.23 ± 7.07 SD) and 59 species in quadrats (mean = 1.47 ± 1.80 SD). In Supplementary Material I, we show that although additional sampling events have the potential to reveal new species, we were able to sample a significant portion of the terrestrial invertebrate community within the system.

### Drivers of terrestrial invertebrate richness

**Fig. 3:**
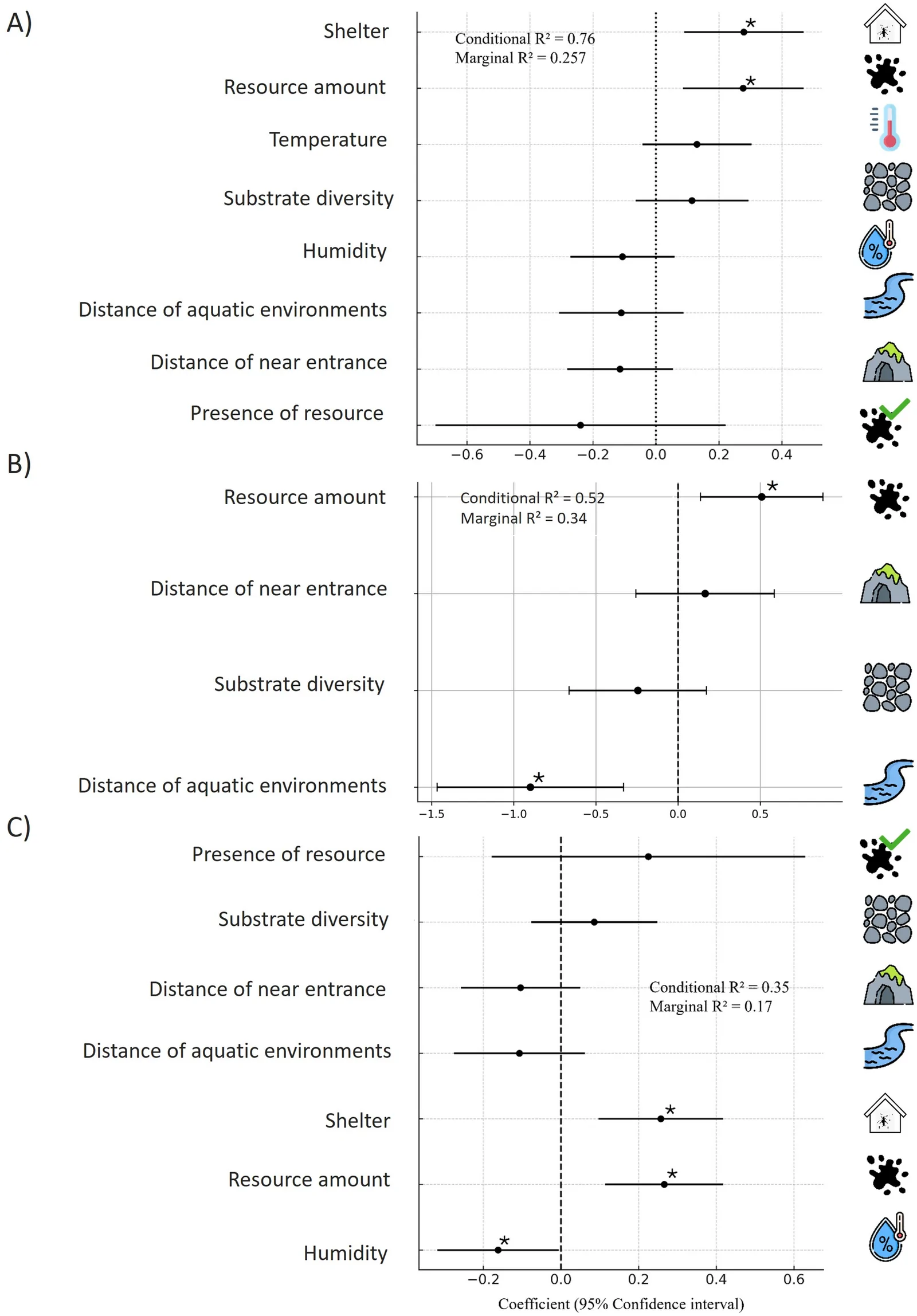
Factors affecting species richness in the Brejões System. **A** invertebrate richness in the sectors; (**B**) troglobite richness in the sectors; (**C**) invertebrate richness in the quadrats. Model average estimated parameters (and 95% confidence interval) for glmm testing the relationship between species richness and predictors variables. (*p < 0.05). Icons made by Freepik from www.flaticon.com

At the mesoscale (sectors), total invertebrate species richness (S) and non-troglobitic species richness (Snt), which were analyzed jointly due to their strong correlation, were positively associated with both resource availability (β ± SE = 0.276 ± 0.095, *p* < 0.01) and shelter availability (β ± SE = 0.278 ± 0.094, *p* < 0.01) (Fig. 3A). No other environmental or spatial predictors exerted significant effects at this scale. The selected model set (Table S2) explained, on average, 70.6% of the total variance in species richness (conditional R²), with 25.7% attributable to fixed effects (marginal R²) and 44.9% to random effects. The full Poisson model met all model assumptions, exhibiting no residual deviations according to *DHARMa* diagnostics (Fig. S6) and low multicollinearity among predictors (VIF < 3; Table S3).

In contrast, troglobitic species richness (St) at the mesoscale was driven by proximity to aquatic environments and resource amount. Specifically, richness declined significantly with increasing distance from aquatic habitats (β ± SE = − 0.899 ± 0.283, *p* = 0.001) (Fig. 3B) and increased significantly with increasing resource availability (β ± SE = 0.509 ± 0.1902, *p* = 0.007). The selected models (Table S4) accounted for an average of 52% of the variance in the data (conditional R²), with fixed effects explaining 34% (marginal R²) and random effects contributing a further 18%. Diagnostic analyses indicated no violations of model assumptions (Fig. S7), no evidence of zero inflation (Fig. S8), and low multicollinearity among predictors (VIF < 4; Table S5).

At the microscale (quadrats), patterns broadly mirrored those observed at the sector level, but with additional complexity. Total invertebrate richness (S) and non-troglobitic richness (Snt) increased significantly with resource availability (β ± SE = 0.266 ± 0.077, *p* < 0.001) and shelter availability (β ± SE = 0.257 ± 0.081, *p* < 0.01) (Fig. 3C). However, relative humidity emerged as an additional significant predictor at this scale, showing a negative relationship with species richness (β ± SE = − 0.162 ± 0.088, *p* = 0.042). The selected model set (Table S6) explained, on average, 35% of the variance in richness (conditional R²), with 17% explained by fixed effects and 18% by random effects. As at the mesoscale, model diagnostics revealed no residual deviations (Fig. S9), no evidence of zero inflation (Fig. S10), and low multicollinearity among predictors (VIF < 3; Table S7).

### Determinants of Beta diversity

At the mesoscale (sectors), total invertebrate β-diversity was structured by a combination of environmental and spatial predictors, whose relative importance ranked as follows: temperature (coef. = 1.64), distance from aquatic environments (coef. = 1.57), resource availability (coef. = 1.56), shelter availability (coef. = 0.61), substrate diversity (coef. = 0.51), distance to the nearest entrance (coef. = 0.24), cave identity (coef. = 0.19), and geographic distance (coef. = 0.13). In contrast, resource presence and humidity did not contribute to explaining total β-diversity. Overall, the model explained 37% of the observed variation; however, permutation tests indicated that only distance from aquatic environments and resource availability were statistically significant predictors, whereas temperature showed a marginal effect (Table 3).

**Table 3:** Relative importance and significance of predictor variables for Beta diversity (Beta total) of terrestrial invertebrates of Brejões System, as determined by permutating 50 times the generalized dissimilarity models (*p < 0.05). We did not perform GDM analyses for troglobitic and non-troglobitic species at the microscale (quadrats)

Variables	Scale	Invertebrates	Non-troglobite	Troglobite
Temperature (°C)	sectors	14.4 (*p* = 0.06)	17.8*	0.8
	quadrats	12.3		
Humidity (%)	sectors			7.9
	quadrats	0.3		
Distance to the near entrance (m)	sectors	0.7	1.5	4.8
	quadrats	0.5		
Distance of aquatic environment (m)	sectors	15.1*	10.5*	18.9*
	quadrats	12.7 (*p* = 0.06)		
Shelter (%)	sectors	5.0	6.5*	4.5
	quadrats	0.2		
Substrate Diversity (H’)	sectors	1.8	3.5	0.4
	quadrats	4.0		
Resource (%)	sectors	12.5*	11.7*	20.3*
	quadrats	28.6*		
Geographic distance	sectors	0.2	0.025	1.0
	quadrats	1.0*		
Cave	sectors	1.0	1.5	
	quadrats			
Presence of resource	sectors			
	quadrats			

**Fig. 4:**
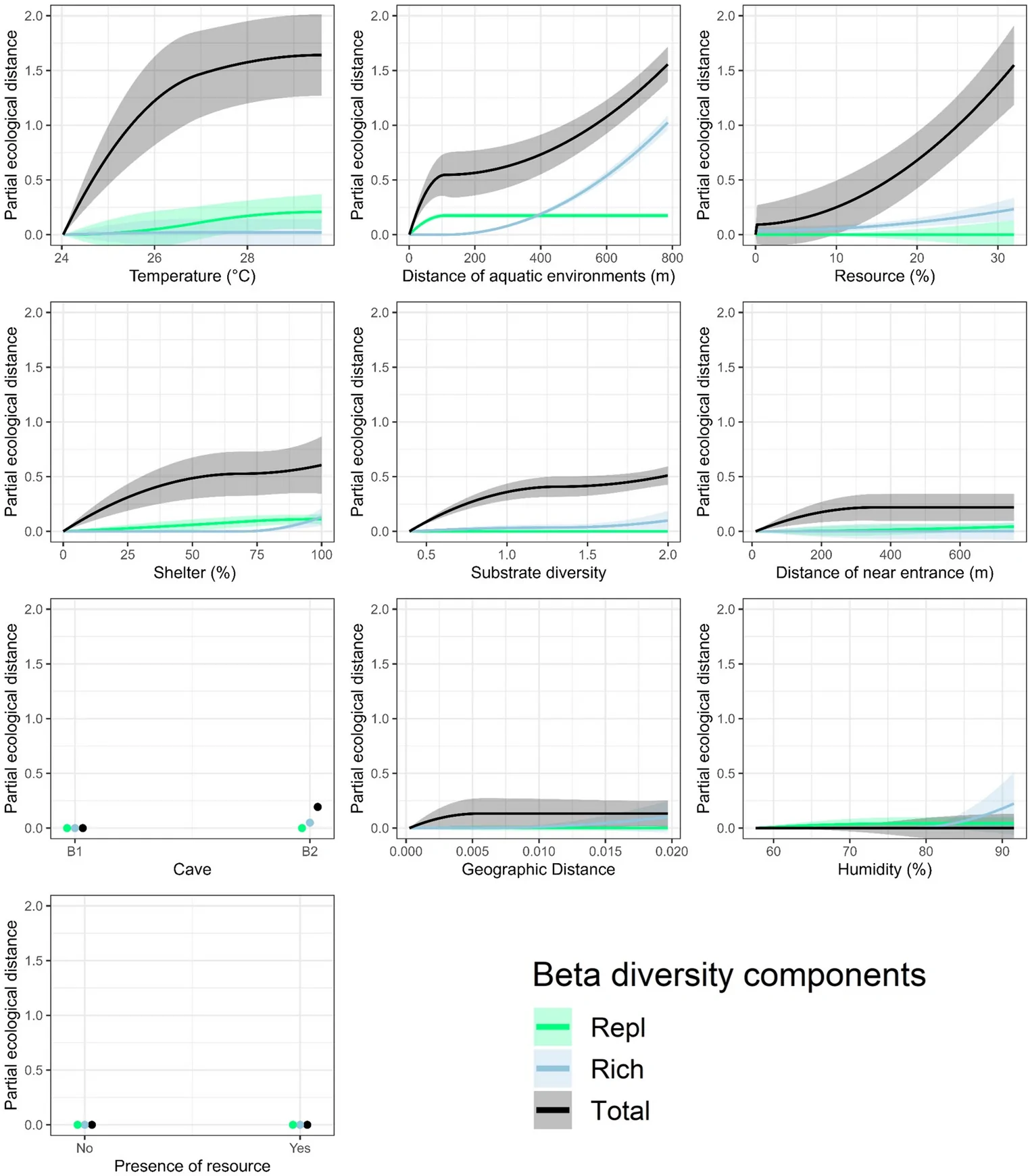
Drivers of invertebrate Beta diversity within the Brejões system using sectors by sampling unit. The maximum height reached by each curve provides an indication of the change of Beta diversity explained by that variable (holding all other variables constant); the larger the value in y, the more influence of the variable. The shape of each spline indicates how the value varies along the gradient (sections of the function with greater inclination imply a greater dissimilarity per unit of the predictive variable). As for the categorical variables, the graphic output is a dot, show difference of beta diversity in the groups. Real geographic distance in meters: 0.000 (0 m), 0.005 (~ 550 m), 0.010 (~ 1100 m), 0.015 (~ 1650 m), 0.020 (~ 2200 m)

The response of β-diversity to temperature followed an exponential pattern without reaching an asymptote and was largely driven by species replacement. Similarly, the distance-to-aquatic-environments gradient exhibited an exponential relationship, with high rates of species turnover up to approximately 100 m, followed by a plateau. Beyond ~ 200 m, changes in β-diversity were increasingly associated with differences in species richness. Resource availability also displayed an exponential relationship without an asymptote, with β-diversity patterns primarily explained by richness differences among sampling units (Fig. 4).

**Fig. 5:**
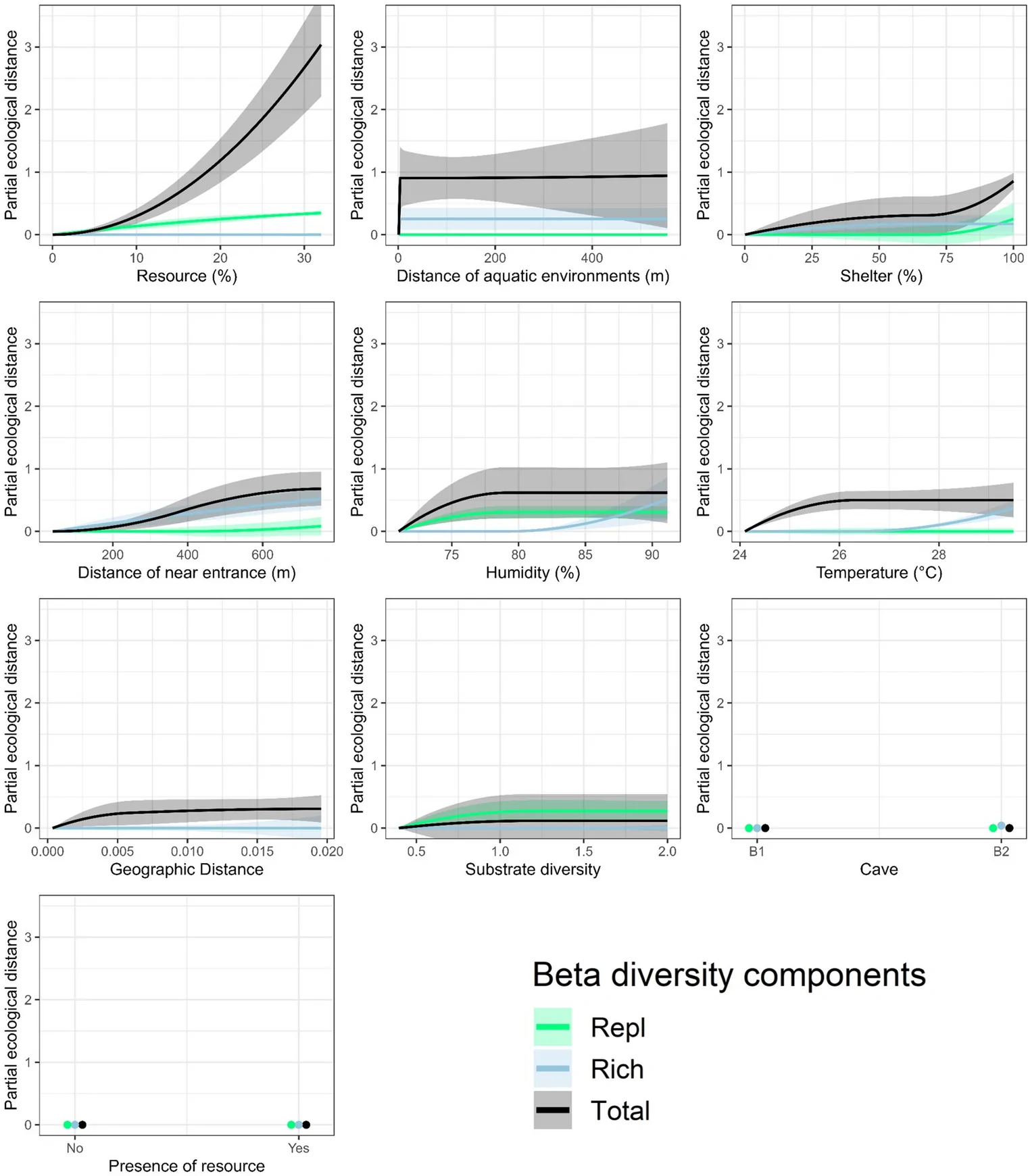
Drivers of troglobite Beta diversity within the Brejões system using sectors by sampling unit. The maximum height reached by each curve provides an indication of the change of Beta diversity explained by that variable (holding all other variables constant); the larger the value in y, the more influence of the variable. The shape of each spline indicates how the value varies along the gradient (sections of the function with greater inclination imply a greater dissimilarity per unit of the predictive variable). As for the categorical variables, the graphic output is a dot, show difference of beta diversity in the groups. Real geographic distance in meters: 0.000 (0 m), 0.005 (~ 550 m), 0.010 (~ 1100 m), 0.015 (~ 1650 m), 0.020 (~ 2200 m)

When analyses were restricted to troglobitic invertebrates at the mesoscale, the relative importance of predictors shifted. Total β-diversity was most strongly associated with resource availability (coef. = 3.07), followed by humidity (coef. = 2.26), distance from aquatic environments (coef. = 0.94), distance to the nearest entrance (coef. = 0.87), shelter availability (coef. = 0.83), temperature (coef. = 0.47), geographic distance (coef. = 0.33), and substrate diversity (coef. = 0.10). In contrast, cave identity and resource presence showed no detectable influence on troglobitic β-diversity. The model explained 40.6% of the observed variance; however, permutation tests indicated that only resource availability and distance from aquatic environments were statistically significant predictors (Table 3). In this context, troglobitic β-diversity responses to distance from aquatic environments were primarily driven by differences in species richness, whereas the relationship with resource availability was approximately linear and largely explained by species replacement. This pattern indicates pronounced compositional turnover along the resource gradient (Fig. 5).

For non-troglobitic invertebrates at the mesoscale, total β-diversity was influenced primarily by temperature (coef. = 1.61), distance from aquatic environments (coef. = 1.29), and resource availability (coef. = 1.18), followed by substrate diversity (coef. = 0.61), shelter availability (coef. = 0.55), distance to the nearest entrance (coef. = 0.29), cave identity (coef. = 0.20), and geographic distance (coef. = 0.05). Humidity and resource presence did not contribute to explaining β-diversity in this group. The model accounted for 32% of the variance, with permutation tests identifying temperature, distance from aquatic environments, resource availability and shelter availability as statistically significant predictors (Table 3).

**Fig. 6:**
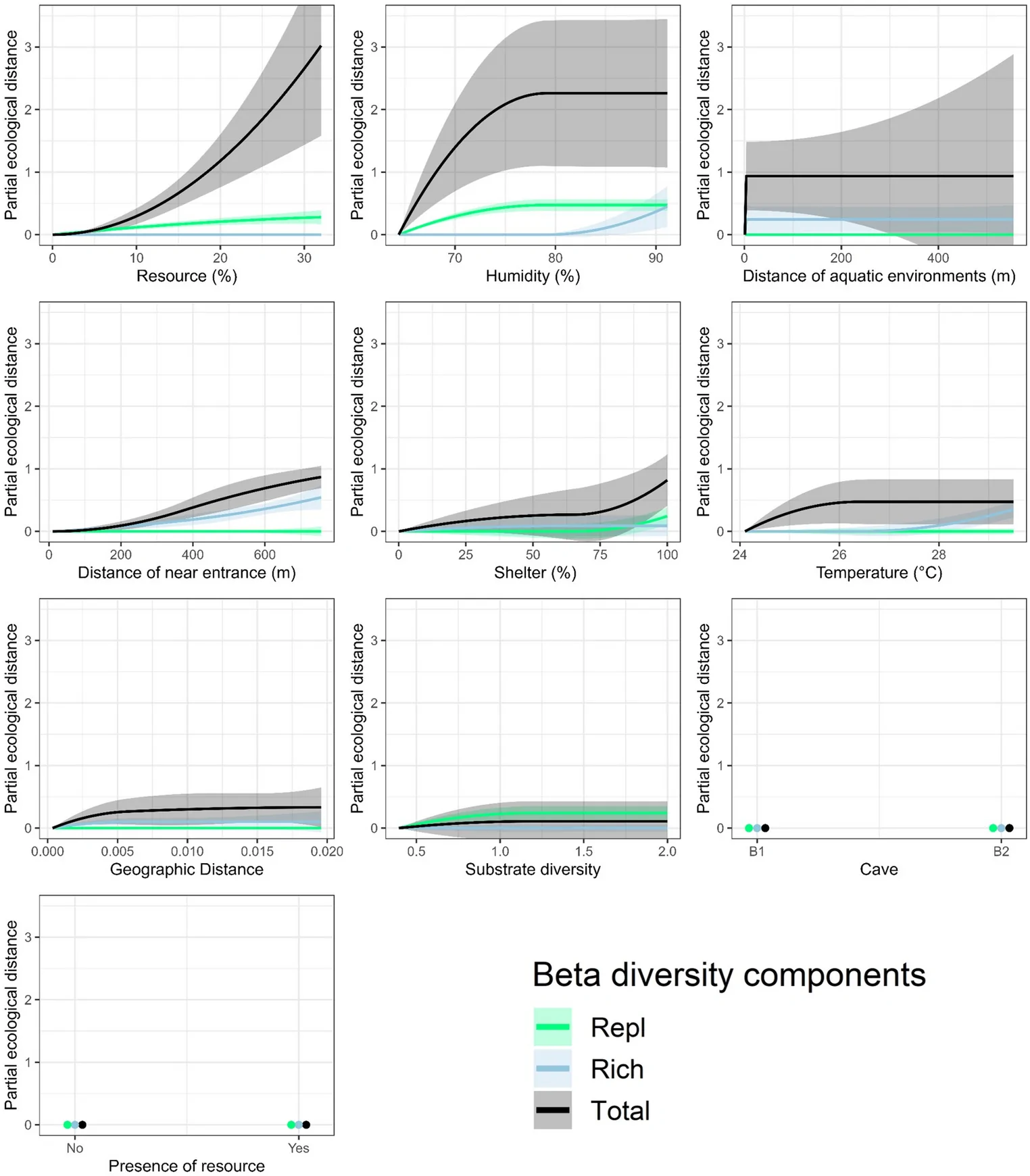
Drivers of non-troglobite Beta diversity within the Brejões system using sectors by sampling unit. The maximum height reached by each curve provides an indication of the change of Beta diversity explained by that variable (holding all other variables constant); the larger the value in y, the more influence of the variable. The shape of each spline indicates how the value varies along the gradient (sections of the function with greater inclination imply a greater dissimilarity per unit of the predictive variable). As for the categorical variables, the graphic output is a dot, show difference of beta diversity in the groups. Geographic distance in meters: 0.000 (0 m), 0.005 (~ 550 m), 0.010 (~ 1100 m), 0.015 (~ 1650 m), 0.020 (~ 2200 m)

The temperature gradient exhibited an exponential relationship without reaching an asymptote and was explained by a combination of richness differences and species replacement. Richness increased with temperature up to approximately 26 °C and then stabilized, while compositional replacement became more pronounced beyond this thermal threshold. The distance to aquatic environments gradient was primarily associated with richness differences, which increased exponentially beyond ~ 200 m. Shelter availability also showed an exponential relationship, stabilizing at approximately 65% surface cover, with β-diversity largely driven by species replacement (Fig. 6).

**Fig. 7:**
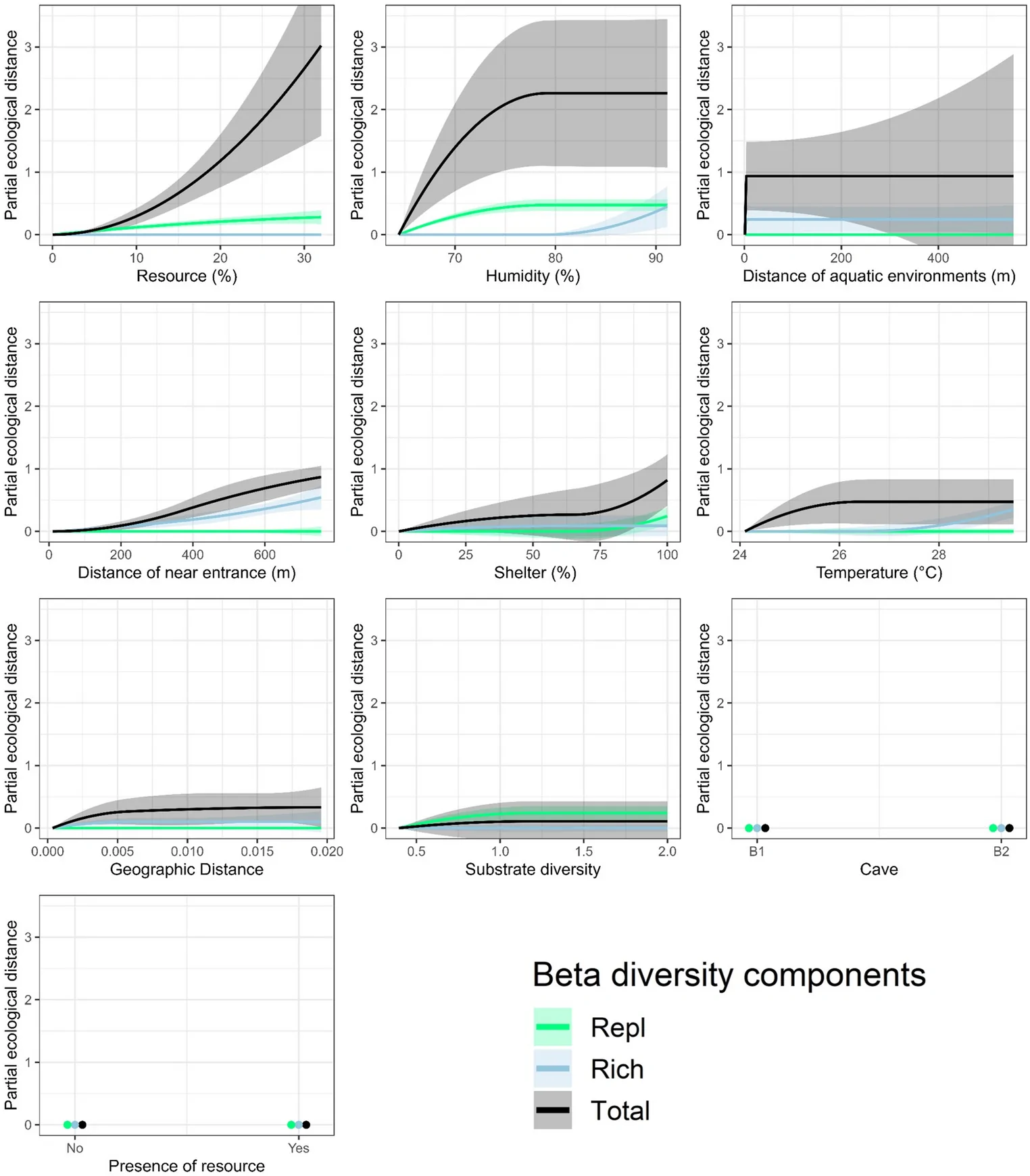
Drivers of invertebrate Beta diversity within the Brejões system using quadrats by sampling unit. The maximum height reached by each curve provides an indication of the change of Beta diversity explained by that variable (holding all other variables constant); the larger the value in y, the more influence of the variable. The shape of each spline indicates how the value varies along the gradient (sections of the function with greater inclination imply a greater dissimilarity per unit of the predictive variable). As for the categorical variables, the graphic output is a dot, show difference of beta diversity in the groups. Real geographic distance in meters: 0.000 (0 m), 0.005 (~ 550 m), 0.010 (~ 1100 m), 0.015 (~ 1650 m), 0.020 (~ 2200 m)

At the microscale (quadrats), generalized dissimilarity modeling revealed a somewhat different ranking of predictors influencing total β-diversity. In descending order of importance, these were temperature (coef. = 2.26), resource availability (coef. = 1.75), distance from aquatic environments (coef. = 1.06), substrate diversity (coef. = 0.93), distance to the nearest entrance (coef. = 0.30), geographic distance (coef. = 0.27), humidity (coef. = 0.09), and shelter availability (coef. = 0.03). Cave identity and resource presence did not contribute to explaining β-diversity at this scale (Fig. 7).

Overall explanatory power at the microscale was substantially lower than at the mesoscale, with the model accounting for only 12.6% of the variance in total β-diversity. Permutation tests indicated that only resource availability and geographic distance were statistically significant predictors, while distance from aquatic environments showed a marginal effect (*p* = 0.06). Despite this, temperature retained high relative importance in the model (Table 3), suggesting a complex but weaker influence on fine-scale compositional turnover.

## Discussion

In this study, we provide comprehensive assessment of the factors structuring terrestrial invertebrate communities within a large, high-volume cave system and the relationship of these factors to neutral and niche-based process. Contrary to our initial expectations, community assembly in the Brejões Cave System was not governed exclusively by environmental filtering but instead reflected a complex interplay between niche-based mechanisms (particularly niche partitioning mediated by resource availability and shelter availability) and, to a lesser extent, neutral processes related to spatial structure. While dispersal-related variables such as geographic distance and distance to cave entrances contributed little to patterns of species dissimilarity, environmental gradients associated with trophic resources, microclimatic stability emerged as key drivers of both species’ richness and β-diversity. Importantly, these effects varied across spatial scales and between troglobitic and non-troglobitic assemblages, highlighting the importance of ecological specialization in mediating community responses.

### Disentangling niche-based and neutral processes in high-volume cave systems

In contrast to our initial hypothesis, environmental filtering did not emerge as the sole or dominant mechanism structuring terrestrial invertebrate communities in the Brejões Cave System. Instead, our results indicate that niche partitioning also plays a substantial role in shaping community composition. As expected, dispersal-related variables associated with neutral processes, such as geographic distance between sampling units and distance to the nearest cave entrance (used here as a proxy for distance from the surface-deep zones under an archipelago island biogeography framework), contributed relatively little to species dissimilarity when compared with predictors linked to environmental filtering and resource-mediated niche differentiation.

Importantly, distance into the cave may also act as a surrogate for environmental gradients rather than solely reflecting dispersal limitation. Previous studies conducted at both individual-cave and regional scales have documented strong associations between increasing distance into the cave and declining organic resource availability, reduced environmental heterogeneity, and greater microclimatic stability (Reis-Venâncio et al. 2024; Souza-Silva et al. 2025; Vaz et al. 2025). However, the strength of these relationships is strongly influenced by cave morphology. In the Brejões Cave System, which contains multiple entrances, we did not detect strong correlations (*r* > 0.7) between distance into the cave and key environmental predictors. This pattern likely reflects enhanced internal connectivity and greater dispersal potential within the system, in contrast to single-entrance caves or systems with entrances aligned along a single axis, where isolation is typically stronger. In such more isolated systems, spatial separation can intensify the structuring of environmental gradients, thereby exerting a stronger influence on species composition and richness (Junta et al. 2025; Souza-Silva et al. 2025) and complicating efforts to disentangle the relative roles of neutral and niche-based processes.

Distance from the aquatic environments (used here as a proxy for microclimatic stability) did not emerge as the dominant driver of β-diversity. Instead, generalized dissimilarity models indicated that trophic resource availability played a role of comparable importance. Previous studies have consistently shown that resource availability strongly influences the distribution and diversity of cave invertebrates (Culver and Pipan 2013; Schneider et al. 2011; Souza-Silva et al. 2011; Pacheco et al. 2020), particularly in dry caves where organic inputs are scarce (Ferreira et al. 2007). Such patterns are often interpreted as the outcome of environmental filtering in oligotrophic systems dominated by generalist taxa, as exemplified by guano communities (Ferreira et al. 2007; Dainelli et al. 2025). However, in the Brejões Cave System, the relationship between β-diversity and resource availability was primarily driven by differences in species richness, suggesting that sites receiving greater resource inputs can sustain a higher number of species through niche partitioning rather than simple environmental exclusion (Pacheco et al. 2020; Vaz et al. 2025).

Proximity to aquatic environments emerged as a key factor shaping terrestrial troglobitic richness. Troglobitic invertebrates typically possess thin cuticles and exhibit limited tolerance to desiccation and thermal variation, rendering them particularly sensitive to microclimatic instability (Colado et al. 2022). As a result, these organisms tend to achieve higher survival and reproductive success in cave sectors characterized by stable, humid, and thermally buffered conditions (Mammola et al. 2020b). In the Brejões System, the course of the Jacaré River corresponds to the lowest portions of the cave, where thermal seasonality is typically reduced (Mammola et al. 2019a). These observations support the use of distance from aquatic environments as a meaningful proxy for climatic stability, allowing it to be interpreted within a niche-based framework.

Consistent with this interpretation, both components of β-diversity contributed to explaining patterns along the distance to water gradient. Species replacement predominated within approximately 100 m of aquatic environments, largely reflecting the occurrence and turnover of troglobitic species. Beyond roughly 200 m, however, variation in β-diversity was driven mainly by differences in species richness. The structuring role of subterranean rivers and pools in shaping cave communities has been reported previously (e.g., Simões et al. 2015; Junta et al. 2025), however, new large-scale studies in the Neotropical region should clarify the potential influence of water bodies on community structuring and the occurrence of troglobitic species.

The factors shaping species richness and community composition in subterranean ecosystems (and their relative importance) are strongly mediated by the degree of specialization to cave life (Junta et al. 2025; Mammola et al. 2019b, 2020b). Accordingly, the relationships between environmental predictors and the ecological processes they represent can differ markedly between troglobitic and non-troglobitic assemblages. In this study, we found that microclimatic variation, particularly temperature gradients ranging from 25 to 29 °C, played a key role in driving species replacement among non-troglobitic invertebrates. This pattern suggests that these taxa differ in their tolerance to microclimatic variability within the cave environment.

This finding is particularly relevant because the category of non-troglobitic species, widely used in ecological analyses of subterranean communities (e.g., Novak et al. 2012; Mammola et al. 2020b), comprises a heterogeneous assemblage that includes troglophiles, trogloxenes, and even accidental species (Novak et al. 2012; Souza-Silva et al. 2021). The absence of consistent and operational criteria for distinguishing among these ecological groups (especially in tropical regions, where taxonomic and ecological knowledge remains limited for most invertebrate species) likely contributes to the wide range of responses observed, particularly in relation to microclimatic sensitivity.

Linear models did not indicate a reduction in non-troglobitic species richness in proximity to aquatic environments. However, generalized dissimilarity models revealed that the β-richness component underlies the relationship between total β-diversity and distance from aquatic habitats. Specifically, differences in species richness increased markedly beyond approximately 250 m from water, indicating a clear spatial structuring of assemblages along this gradient that was not captured by linear richness models alone.

Beyond microclimatic effects, resource availability and shelter availability also influenced the assembly of non-troglobitic communities. Cave sectors characterized by higher resource inputs and greater shelter availability supported the coexistence of a larger number of species, consistent with classic niche theory predicting reduced competitive exclusion through niche differentiation (MacArthur and Levins 1967). In contrast, troglobitic assemblages exhibited a markedly different response: increased resource availability was primarily associated with species replacement rather than increases in richness. This pattern may reflect fundamental differences in ecological strategies between strictly subterranean species and facultative cave dwellers, particularly in their capacity to cope with temporal variability in resource supply.

Recent evidence suggests that morphological traits associated with subterranean adaptation (such as eye and pigment reduction) do not necessarily coevolve with metabolic adaptations or thermal tolerance linked to energy economy (Kozel et al. 2023). This decoupling implies that troglobitic species may exhibit distinct and potentially narrow nutritional requirements, which could help explain the observed patterns of species turnover along resource gradients.

It is important to note that, in this study, we assumed that geographic distance is associated with neutral processes, particularly those related to dispersal, an interpretation that has been widely debated in the ecological literature (Vellend et al. 2014). Despite these ongoing discussions, our results indicate that this variable does not exert a significant influence on the assembly of terrestrial invertebrate communities in the Brejões System. We further acknowledge that temporal dynamics were not assessed due to logistical and funding constraints. This limitation may lead to an underestimation of the role of neutral processes and should therefore be considered when interpreting the results.

### Scale-dependent drivers of biodiversity

As expected, comparisons between the two spatial scales analyzed (30 m² sectors and 1 m² quadrats) revealed clear scale-dependent differences in the factors influencing invertebrate species richness and community composition. Similar patterns have been documented in previous studies employing comparable methodological frameworks (Junta et al. 2025; Rocha Melo et al. 2026; Souza-Silva et al. 2025 ). At the microscale (quadrats), total species richness (troglobitic and non-troglobitic combined) was primarily influenced by resource availability, shelter availability, and humidity. In contrast, β-diversity at this scale was associated with resource availability and geographic distance, suggesting a modest contribution of neutral processes to community assembly. At the mesoscale (sectors), species richness was similarly driven by resource availability and shelter availability. However, β-diversity was primarily influenced by distance from aquatic environments and resource availability, indicating a stronger role of niche-based processes in structuring communities at this broader spatial scale.

Relative humidity exerted a negative effect on species richness only at the microscale, whereas the positive influence of resource availability and shelter availability was consistent across both sampling scales. In Romanian caves, sites with humidity levels approaching saturation have been associated with reduced arthropod species richness, suggesting that near-saturated conditions impose physiological constraints that limit the number of species able to persist (Bǎncilǎ et al. 2026). In our study, an examination of the relationship between the richness-difference component (Brich) and humidity at the microscale revealed a noticeable pattern between 80% and 90% relative humidity (Fig. 7), potentially indicating zones of reduced species richness. Although broadly consistent with our findings, the study by Bǎncilǎ et al. (2026) employed a different sampling design, based on 100 m transects and cave wall surveys, which may influence the comparability of results.

Scale dependence was also evident in the drivers of β-diversity. A distance–decay pattern, characterized by decreasing compositional similarity with increasing geographic distance (Nekola and White 1999), was detected exclusively at the microscale, although its overall contribution was relatively weak. This observation may reflect incomplete detection of species distributions across the system at finer spatial resolutions, leading to greater apparent dissimilarity among quadrats. As the sampling unit increases in size, a larger number of species is captured, and their spatial distributions are likely more accurately represented.

It is important to note, however, that the Brejões Cave System is characterized by exceptionally large conduits, a condition not shared by all subterranean systems. Consequently, the use of small sampling units such as 1 m² quadrats remains a common and appropriate approach in cave ecology studies (Dainelli et al. 2025; Mammola et al. 2021; Pacheco et al. 2025), particularly in systems with more restricted spatial dimensions.

Taken together, these results indicate that ecological patterns, processes, and inferred mechanisms are sensitive to sampling scale and data resolution. This highlights the importance of carefully designed, scale-explicit methodologies when monitoring fauna and interpreting biodiversity patterns in large cave systems.

### Functional connectivity within the Brejões cave system

The Brejões karst region comprises a ~ 120 m deep canyon formed by the collapse of a former northern section of the Brejões cave, shaping the current cave system configuration (Furtado et al. 2022). We assessed whether this configuration influences β-diversity patterns. Cave identity had a negligible effect in generalized dissimilarity models, and geographic distance showed consistently weak influence. Species dissimilarity patterns were mainly driven by richness differences, suggesting that Brejões II represents a subset of Brejões I for both troglobitic and non-troglobitic assemblages. This pattern was stronger for troglobitic species, which typically have restricted distributions. Few troglobitic taxa were shared between caves, and overlap decreased further under standardized sampling.

Despite limitations due to incomplete sampling, common in subterranean studies, our results indicate functional connectivity between cave segments. The Jacaré River and the karst fracture network (Furtado et al. 2022) likely facilitate invertebrate dispersal. Future population and genetic studies are needed to test this hypothesis, particularly given the prevalence of cryptic species. Overall, the Brejões Cave System represents a valuable natural laboratory for studying evolutionary and biogeographic processes in subterranean ecosystems.

### Conservation perspectives

Our results demonstrate that the Brejões Cave System harbors an exceptionally rich and distinctive invertebrate fauna, including numerous species (and even genera) that remain undescribed. Although it harbors 23 troglobitic species rather than the 25 proposed as the threshold for classification as a hotspot according to Culver and Pipan (2013), we consider the Brejões System a Hotspot of Subterranean Biodiversity. Recent studies have questioned the use of a fixed threshold, advocating instead for a more flexible framework that incorporates contextual factors such as geographic setting, lithology, and biome, thereby providing a more ecologically meaningful assessment of subterranean biodiversity value (Ferreira et al. 2023). Furthermore, when considering the original definition of HSB, which established a threshold of 20 cave-restricted species, the Brejões System clearly meets (and exceeds) this criterion.

The system represents the sixth limestone Hotspot of Subterranean Biodiversity identified in South America (see Junta et al. 2025; Souza-Silva and Ferreira 2016; Souza-Silva et al. 2021; Vaz et al. 2025), five of which occur within the Caatinga domain (seasonally dry tropical forest). This striking concentration of subterranean biodiversity supports the hypothesis that historical climatic fluctuations in the region promoted population isolation within caves, thereby fostering high levels of endemism (Ferreira et al. 2023).

Despite being located within an Environmental Protection Area, recent studies have detected the presence of heavy metals in sediments from subterranean water pools and in the Jacaré River (Vaz, Souza-Silva and Ferreira 2025). Although current concentrations fall within limits permitted by Brazilian environmental legislation, potential ecological impacts cannot be dismissed. Troglobitic species are particularly vulnerable due to their low metabolic rates, extended lifespans, and reduced production of metallothioneins, which are proteins involved in the detoxification and elimination of heavy metals (Dickson et al. 1979). These physiological traits increase the risk of metal bioaccumulation, which has been recognized as a significant threat in subterranean ecosystems (Dickson et al. 1979).

Furthermore, the designation of protected areas does not shield subterranean ecosystems from the effects of climate change (Sánchez-Fernández et al. 2021). Given that microclimatic stability emerged as a key factor structuring invertebrate communities in the Brejões System, the implementation of long-term monitoring programs is essential to support adaptive management and conservation planning.

## Supplementary Information

Supplementary Material 1.

Supplementary Material 2.

## Data Availability

The datasets supporting the conclusions of this article are available in the repository figshare, https://doi.org/10.6084/m9.figshare.31864222.
