# Supplementary material for: Drivers of invertebrate community structure in a high-volume Neotropical cave system: a newly recognized subterranean biodiversity hotspot
Source: Oecologia. 2026 Oct 16;208(11):151. doi: 10.1007/s00442-026-05989-0 (PMC13675341; doi:10.1007/s00442-026-05989-0)
Supplement: Supplementary file 1 [file 442_2026_5989_MOESM1_ESM.docx]

**Drivers of invertebrate community structure in a high-volume Neotropical cave system: a newly recognized subterranean biodiversity hotspot**

Original Article

Gabriel Augusto Silva Vaz^a,b^, Rodrigo Lopes Ferreira^a,b^ Marconi Souza Silva^a,b^

^a^Programa de Pós-graduação em Ecologia Aplicada, Universidade Federal de Lavras, Lavras, Minas Gerais, Brasil, CEP 37200-900.

^b^Centro de Estudos em Biologia Subterrânea, Departamento de Ecologia e Conservação, Instituto de Ciências Naturais, Universidade Federal de Lavras, Lavras, Minas Gerais, Brasil, CEP 37200-900.

*Corresponding author: Gabriel Augusto Silva Vaz (gabrielpeixevaz@gmail.com)

**Supplementary Material 1**

**Material and Methods**

***Study area***

The system consists of a series of subparallel conduits oriented predominantly from south to north, associated with both ancient and currently active courses of the subterranean Jacaré River (Berbert-Born and Karmann 2002; Furtado et al., 2022). Several conduits exceed 40 m in height and 50 m in width, and the main entrance reaches more than 100 m in height (Berbert-Born and Karmann 2002; Furtado et al. 2022; Fig. 1).

At present, the river flows through the western conduits, where phreatic pools are also present. During flood events, these pools become hydraulically connected to the Jacaré River (Vaz, Souza-Silva and Ferreira 2025b), corresponding to the lowest sectors of the cave system (Furtado et al. 2022). In contrast, conduits lacking active flow are predominantly dry and characterized by clastic deposits, including large collapse blocks, as well as well-developed speleothems and ceiling openings (skylights) that allow light penetration and the input of surface runoff into the cave (Berbert-Born and Karmann 2002; Furtado et al. 2022; Fig. 1, and Fig. S1 in Supplementary Material 1).

The Jacaré River is a perennial tributary of the São Francisco River and sustains comparatively more mesic vegetation along its course throughout the year relative to the surrounding semi-arid landscape (Berbert-Born and Karmann 2002). Its long-term erosive activity has carved a canyon exceeding 100 m in depth in certain karst sectors, largely as a result of the collapse of ancient cave conduits (Berbert-Born and Karmann 2002; Fig. S1). Rather than entering Brejões Cave I through its main entrance, the Jacaré River sinks approximately 300 m upstream of the cave, reemerging inside the cave at a lower level roughly 750 m from the main entrance. From this point, the river flows through portions of the lower galleries, traversing the westernmost conduit before sinking again near the northern sector of the lower chamber, in the vicinity of the second skylight. The watercourse subsequently resurges within Brejões Cave II, where it flows through most of the western conduit and ultimately emerges into the external environment near the northernmost entrance of that cave.

***Predictors in different scales***

It is important to note that certain substrate types, such as Large Rock (1000–4000 mm; LR), Medium Rock (500–1000 mm; MR), and Small Rock (250–500 mm; SR), may function differently depending on the spatial scale considered. When analyzed at the microscale (quadrats), these substrates may act as general substrates rather than providing shelter. Accordingly, during photograph-based classification and quantification at the quadrat level, rock substrates were reclassified as Rough Rock (RR) or Smooth Rock (SMR), reflecting their functional role at this scale.

***Identifying Potential Troglobites***

Troglobites are organisms that are restricted to caves or other shallow subterranean habitats and are unable to maintain permanent populations in surface ecosystems. Typical troglomorphic features include reduced or absent eyes and pigmentation, as well as elongated sensory and/or locomotory appendages (Sket 2008; Deharveng et al. 2024; Zampaulo, Souza-Silva and Ferreira 2025). In many subterranean taxa, these traits are often accompanied by increased body size and the development of enhanced sensory structures, reflecting adaptations to the dark and resource-limited cave environment (Sket 2008; Deharveng et al. 2024; Zampaulo, Souza-Silva and Ferreira).

However, troglomorphy is not uniformly expressed across all troglobites. The degree of morphological specialization may vary according to habitat size, light availability in twilight zones, genetic variability, and other ecological and evolutionary processes. This variability has led to the recognition of eutroglophile species, which are strongly associated with subterranean environments but do not exhibit pronounced troglomorphic traits (Sket 2008; Deharveng et al. 2024). Accordingly, a few taxa in the present study are conservatively classified as potential troglobites, ensuring terminological consistency until more detailed ecological, behavioral, and genetic analyses allow a more definitive assessment of their subterranean dependence.

To further support the recognition of troglomorphic characteristics, taxonomic specialists with expertise in specific groups were consulted during specimen identification (see Acknowledgements). This expert input contributed to the accurate evaluation of morphological traits indicative of subterranean adaptation (Furtado-Oliveira et al. 2022; Souza-Silva, Lourenço-Guimarães and Ferreira 2025).

**Data analysis**

***Exploratory analysis***

To assess significant differences in invertebrate species richness/non-troglobitic species richness, troglobitic species richness, temperature, humidity, shelter/general substrates, presence of resource, and substrate diversity/shelter diversity between the margins of the aquatic environments (represented by sectors 1, 3, 4, 5, 6, 11, 12, 13, 14, 15, 39, and 40 in Brejões I, and sectors 3, 4, 7, and 10 in Brejões II) and the remaining regions of the Brejões Cave System, we used generalized linear models (GLMs).

Differences between these groups were assessed using generalized linear models (GLMs). Models were fitted using the glmmTMB package (Brooks et al. 2017; https://CRAN.R-project.org/package=glmmTMB), with Poisson and negative binomial (nbinom2) error distributions applied to richness variables, and Gaussian error distributions applied to the remaining continuous predictors. Model assumptions and residual diagnostics were evaluated using the simulateResiduals function from the DHARMa package (https://CRAN.R-project.org/package=DHARMa).

Because the variable resource presence did not meet the assumptions required for Gaussian modeling, differences between aquatic and non-aquatic sectors for this variable were assessed using the nonparametric Kruskal–Wallis test, which evaluates differences in median values between groups.

***Variations in species richness and environmental variables among cave habitats***

Troglobitic species richness differed markedly among cave habitats, with shoreline sectors harboring significantly higher numbers of troglobitic species than other regions of the system. This pattern was consistent at both spatial scales (sectors: β ± SE = 1.90 ± 0.37, *p* < 0.0001, R² = 0.32; quadrats: β ± SE = 2.44 ± 0.49, *p* < 0.0001, R² = 0.17; Fig. S5). In contrast, no significant differences among habitats were detected for total invertebrate richness (i.e., troglobitic + non-troglobitic species) at either the sector or quadrat scale (sectors: β ± SE = 0.22 ± 0.20, *p* = 0.275; quadrats: β ± SE = 0.09 ± 0.17, *p* = 0.591).

Environmental conditions also differed consistently between shoreline and non-shoreline habitats. Shoreline sectors were characterized by significantly lower temperatures (sectors: β ± SE = −1.47 ± 0.32, *p* < 0.0001, R² = 0.28; quadrats: β ± SE = −1.47 ± 0.19, *p* < 0.0001, R² = 0.26) and higher relative humidity (sectors: β ± SE = 6.23 ± 2.19, *p* < 0.0001, R² = 0.13; quadrats: β ± SE = 6.23 ± 1.28, *p* < 0.0001, R² = 0.12) compared to other cave habitats (Fig. S5).

Differences in resource availability and shelter varied with spatial scale. At the sector level, neither resource presence nor shelter availability differed significantly between habitats (resource: χ² = 3.13, *p* = 0.076; shelter: β ± SE = −7.13 ± 0.36, *p* = 0.439). In contrast, when quadrats were used as the sampling unit, both variables differed significantly between habitats (resource: χ² = 8.41, *p* = 0.004; shelter: β ± SE = 13.45 ± 6.37, *p* = 0.034, R² = 0.03). Specifically, shoreline quadrats exhibited higher resource availability and lower shelter availability, which consequently corresponded to higher availability of general substrates, given the strong negative correlation between shelter and general substrate cover (Fig. S5). Nevertheless, these patterns were characterized by substantial within-habitat variability and relatively low explanatory power.

Substrate diversity (and its collinear counterpart, shelter diversity) did not differ significantly between shoreline and non-shoreline habitats at either spatial scale (sectors: β ± SE = −0.17 ± 0.13, *p* = 0.199; quadrats: β ± SE = −0.01 ± 0.07, *p* = 0.861).

Finally, sites where trophic resources were present exhibited consistently cooler and more humid conditions than sites lacking detectable resources. This pattern was observed at both the sector (temperature: β ± SE = −0.79 ± 0.33, *p* = 0.016, R² = 0.10; humidity: β ± SE = 5.75 ± 2.01, *p* = 0.044, R² = 0.13) and quadrat scales (temperature: β ± SE = −0.52 ± 0.21, *p* = 0.014, R² = 0.04; humidity: β ± SE = 3.74 ± 1.31, *p* = 0.004, R² = 0.04; Fig. S5).

**Rarefaction/extrapolation curves of species richness and sample coverage**

Using sampling data of January 2024, we performed rarefaction/ extrapolation curves of species richness (troglobites, non-troglobites and both categories) and sample coverage for mesoscale and microscale. All analyses followed the framework of Hsieh, Ma and Chao (2016), implemented using iNEXT package, with richness extrapolated to twice the reference sampling effort (in terms of both individuals and sampling units).

Our rarefaction and extrapolation curves using differences metrics (number of individuals – Figure S11 A and C and number of sampling units – Figure S12 A and C) for terrestrial non-troglobite species, terrestrial troglobite species and for both ecological categories combined species richness continues to increase with increased sampling effort. The patterns are consistent across both spatial scales analyzed, however the mesoscale extrapolation estimated more total species with microscale (Figure S11 A and C; Figure S12 A and C).

Additionally, at both spatial scales coverage based on abundance data reached approximately ~90% (Figure S11 B and D), whereas coverage based on sampling units was approximately ~80% (Figure S12 B and D). These results indicate that additional sampling events could still reveal new species. Nevertheless, the achieved sampling coverage is substantial and sufficient to support robust inferences regarding the factors influencing species richness and composition along the Brejões Cave System.

Fig S1. (A) Aerial photograph illustrating the extent of the fluviokarst system containing the caves of the Brejões complex. The yellow arrow indicates the main entrance of Brejões I Cave, and the white arrow indicates a skylight. The quadrilaterals shown in the upper right corner delimit the areas presented in panels (B) and (C). In panel (B), the yellow arrow indicates the location of the downstream entrance of Brejões I Cave. (B) Canyon located near the central portion of the Brejões Cave System. The yellow arrow indicates the downstream

Table S1 - Details about how each substrate were linked each categories

| **Variables** | **Abbreviation** | **Unit Meansurement** | **Description** | **Categories** |
| --- | --- | --- | --- | --- |
| Guano | GU |  | Bat feces | Trophic resource |
| Feces | FC |  | Feces of no-flying animals | Trophic resource |
| Carcasses | CRC |  |  | Trophic resource |
| Roots | ROO |  |  | Trophic resource |
| Leaf Litter (serraprilheira) | LL | > 10 mm |  | Trophic/Shelter |
| Plant Debris (detrito vegetal) | PD | < 10 mm |  | Trophic resource |
| Fine Branch (galho fino) | FB | 11-30 mm |  | Trophic/Shelter |
| Medium Branch (galho médio) | MB | 31-50 mm |  | Trophic/Shelter |
| Coarse Branch (galho grosso) | CBR | 51-250 mm |  | Trophic/Shelter |
| Trunk (tronco) | TR | > 250 mm |  | Trophic/Shelter |
| Watercourse (curso d'água) | WC |  |  | General |
| Water Pond (poça de água) | WP |  |  | General |
| Drip Water (gotejamento de água) | DP |  |  | General |
| Termite Hills (termiteiros) | TH |  |  | General |
| Cryptogams | CTG |  | Bryophytes and Ferns | Trophic resource |
| Phanerogams | PNG |  | Monocotyledons and Dicotyledons | Trophic resource |
| Algae | AL |  |  | Trophic resource |
| Actinomycetes | ACT |  |  | Trophic resource |
| Basidiomycetes | BAM |  |  | Trophic resource |
| Another organic substrate | AOS |  | Such Feathers | Trophic resource |
| Rough Rock (rocha rugosa) | RR |  |  | General |
| Smooth Rock (rocha lisa) | SMR |  |  | General |
| Large Rock (matacão largo) | LR | 1000-4000 mm |  | Shelter |
| Medium Rock (matacão médio) | MR | 500-1000 mm |  | Shelter |
| Small Rock (matacão pequeno) | SR | 250-500 mm |  | Shelter |
| Cobbles (Bloco) | CB | 64 to 250 mm |  | Shelter |
| Coarse gravel (cascalho grosso) | CG | 16 to 64 mm |  | Shelter |
| Fine gravel (casgalho fino) | FG | 2 to 16 mm |  | Shelter |
| Sand | SAN | 0,06 to 2 mm |  | General |
| Silte | SIL | < 0,05 mm |  | General |
| Hardpan | HRP |  | consolidated clay | General |
| Retraction Cracks | RC |  | spaces between Hardpan | Shelter |
| Calcite Rafts | RT |  | Type of speleothems | Shelter |
| Stalagmite | STG |  | Type of speleothems | General |
| Travertine | TRA |  | Type of speleothems | General |
| Flowstone | FLO |  | Type of speleothems | General |
| Cauliflower speleothems | CF |  | Type of speleothems | General |
| Another inorganic substrates | AIS |  | Such trash | General |
| Temperature | Temp | C° |  | Microclimatic |
| Humidity | HUM | kg/m³ |  | Microclimatic |
| Distance from the near  aquactic environments | DA | m | Approximate measurement obtained from cave maps (not accounting for elevation differences). | Physical |
| Distance from the  near entrance | DE | m | Approximate measurement obtained from cave maps (not accounting for elevation differences). | Physical |
| Availability of shelter | shelter | % | sum of proportions of shelter substrates | Shelter |
| Availability of resource | resource | % | sum of proportions of Trophic resource | Trophic resource |
| Availability of  general substrate | general | % | sum of proportions of general substrates (Substrates that are unlikely to serve as shelters for invertebrates) | General |
| Shelter diversity | shelter H' |  | Shannon-Weaver Index (using the proportions of shelter substrates) | Environmmental  Heterogeneity |
| Resource diversity | resource H' |  | Shannon-Weaver Index (using the proportions of Trophic resource) | Environmmental  Heterogeneity |
| Substrate diversity | subs H' |  | Shannon-Weaver Index all substrates | Environmmental  Heterogeneity |

A)

B)

Fig S2. Mean substrate values for each category observed at the mesoscale (sectors) (A) and microscale (quadrats) (B).

Fig S3. Correlation between variables at the mesoscale (sectors); temperature (temp), Humidity (HUM), number of collectors, start time, spent time, Distance of near entrance (DE), Distance of near aquatic environment (DA), Habitat (near aquatic (A), far aquatic (FA)), shelter, resource, presence of resource, general, shelter diversity (shelter_H), resource diversity (resource_H), general diversity (general_H), substrate diversity (subs_H), terrestrial invertebrate richness (S), terrestrial troglobitic invertebrate richness(S_t), terrestrial non-troglobitic invertebrate richness (S_nt).

Fig S4. Correlation between variables at the microscale (quadrats): temperature (temp), Humidity (HUM), number of collectors, start time, spent time, Distance of near entrance (DE), Distance of near aquatic environment (DA), Habitat (near aquatic (A), far aquatic (FA)), shelter, resource, presence of resource, general, shelter diversity (shelter_H), resource diversity (resource_H), general diversity (general_H), substrate diversity (subs_H), terrestrial invertebrate richness (S), terrestrial troglobitic invertebrate richness(S_t), terrestrial non-troglobitic invertebrate richness (S_nt).

Fig S5. Differences between the habitats of the Brejões System. A: sampling units along the shoreline habitats; FA: sampling units in the other environments of the system; Black points: mean of each group.

Table S2. Best models for invertebrate richness in the sectors. Model selection according to the corrected Akaike criterion for small sampling size. We only listed the models with delta < 2. AICc, Corrected Akaike information criterion for small sample size; delta AICc, (AIC of the model - AIC of the best model); Akaike weight; Marginal R², variance explained by the fixed effects alone; Conditional R², variance explained by both the fixed and random effects together.

| cond(DA) | cond(DE) | cond(HUM) | cond(p_resource) | cond(resource) | cond(shelter) | cond(subs_H) | cond(temp) | df | AICc | delta | Weight | Marginal R² | Conditional R² |
| --- | --- | --- | --- | --- | --- | --- | --- | --- | --- | --- | --- | --- | --- |
|  |  |  |  | 0.268 | 0.303 |  | 0.128 | 5 | 322.841 | 0.000 | 0.099 | 0.254 | 0.708 |
|  |  |  |  | 0.244 | 0.301 |  |  | 4 | 322.862 | 0.021 | 0.098 | 0.216 | 0.707 |
|  | -0.127 |  |  | 0.256 | 0.271 |  |  | 5 | 322.896 | 0.055 | 0.096 | 0.253 | 0.703 |
|  |  | -0.117 |  | 0.268 | 0.297 |  |  | 5 | 323.229 | 0.389 | 0.081 | 0.247 | 0.706 |
|  |  |  |  | 0.246 | 0.257 | 0.122 |  | 5 | 323.343 | 0.503 | 0.077 | 0.246 | 0.706 |
|  | -0.110 |  |  | 0.274 | 0.276 |  | 0.110 | 6 | 323.545 | 0.705 | 0.069 | 0.278 | 0.703 |
|  |  |  |  | 0.268 | 0.263 | 0.109 | 0.117 | 6 | 323.753 | 0.913 | 0.063 | 0.276 | 0.707 |
|  |  |  | + | 0.332 | 0.307 |  |  | 5 | 323.955 | 1.114 | 0.057 | 0.237 | 0.708 |
|  |  |  | + | 0.349 | 0.259 | 0.137 |  | 6 | 324.020 | 1.180 | 0.055 | 0.275 | 0.709 |
|  |  | -0.108 |  | 0.267 | 0.257 | 0.112 |  | 6 | 324.046 | 1.205 | 0.054 | 0.272 | 0.705 |
| -0.099 |  |  |  | 0.285 | 0.278 |  | 0.172 | 6 | 324.337 | 1.496 | 0.047 | 0.270 | 0.710 |
|  | -0.099 | -0.083 |  | 0.270 | 0.275 |  |  | 6 | 324.441 | 1.601 | 0.044 | 0.266 | 0.703 |
| -0.123 | -0.126 |  |  | 0.296 | 0.242 |  | 0.162 | 7 | 324.569 | 1.728 | 0.042 | 0.302 | 0.706 |
|  | -0.114 |  | + | 0.321 | 0.279 |  |  | 6 | 324.619 | 1.778 | 0.041 | 0.264 | 0.704 |
|  | -0.095 |  |  | 0.254 | 0.251 | 0.077 |  | 6 | 324.758 | 1.917 | 0.038 | 0.262 | 0.703 |
|  |  |  | + | 0.322 | 0.306 |  | 0.109 | 6 | 324.827 | 1.987 | 0.036 | 0.260 | 0.708 |

Figure S6. Results of DHARMa residual for the Poisson GLMM of invertebrate richness across sectors. Left panel creates a qq-plot to detect overall deviations from the expected distribution, by default with added tests for correct distribution (KS test), dispersion and outliers (Hartig, 2024; https://cran.r-project.org/web/packages/DHARMa/vignettes/DHARMa.html).

Table S3. Variance inflation factors (VIFs) for the Poisson GLMM of invertebrate richness across sectors.

| Term | VIF | VIF 95% CI | Increased SE | Tolerance | Tolerance 95% CI |
| --- | --- | --- | --- | --- | --- |
| temp | 2.12 | [1.61, 3.05] | 1.46 | 0.47 | [0.33, 0.62] |
| HUM | 1.86 | [1.44, 2.66] | 1.36 | 0.54 | [0.38, 0.69] |
| DE | 1.69 | [1.34, 2.43] | 1.30 | 0.59 | [0.41, 0.75] |
| DA | 1.51 | [1.22, 2.17] | 1.23 | 0.66 | [0.46, 0.82] |
| Shelter | 1.44 | [1.18, 2.08] | 1.20 | 0.70 | [0.48, 0.85] |
| resource | 2.23 | [1.68, 3.22] | 1.49 | 0.45 | [0.31, 0.59] |
| P_resource | 2.39 | [1.79, 3.46] | 1.55 | 0.42 | [0.29, 0.56] |
| Subs_H | 1.57 | [1.26, 2.25] | 1.25 | 0.64 | [0.44, 0.79] |

Table S4. Best models for troglobitic richness in the sectors. Model selection according to the corrected Akaike criterion for small sampling size. We only listed the models with delta < 2. AICc, Corrected Akaike information criterion for small sample size; delta AICc, (AIC of the model - AIC of the best model); Akaike weight; Marginal R², variance explained by the fixed effects alone; Conditional R², variance explained by both the fixed and random effects together.

| cond(DA) | cond(DE) | cond(HUM) | cond(p_resource) | cond(resource) | cond(shelter) | cond(subs_H) | cond(temp) | df | AICc | delta | weight | Marginal R² | Conditional R² |
| --- | --- | --- | --- | --- | --- | --- | --- | --- | --- | --- | --- | --- | --- |
| -0.924 |  |  |  | 0.532 |  |  |  | 4 | 126.400 | 0.000 | 0.500 | 0.346 | 0.534 |
| -0.882 |  |  |  | 0.483 |  | -0.244 |  | 5 | 127.443 | 1.043 | 0.297 | 0.342 | 0.532 |
| -0.859 | 0.1644 |  |  | 0.488 |  |  |  | 5 | 128.21 | 1.817 | 0.201 | 0.341 | 0.515 |

Figure S7. Results of DHARMa residual for the Poisson GLMM of troglobite richness across sectors. Left panel creates a qq-plot to detect overall deviations from the expected distribution. by default with added tests for correct distribution (KS test). dispersion and outliers (Hartig. 2024; https://cran.r-project.org/web/packages/DHARMa/vignettes/DHARMa.html).

Figure S8. Result of DHARMa zero-inflation test for the Poisson GLMM of troglobite richness across sectors.

Table S5. Variance inflation factors (VIFs) for the Poisson GLMM of troglobite richness across sectors.

| Term | VIF | VIF 95% CI | Increased SE | Tolerance | Tolerance 95% CI |
| --- | --- | --- | --- | --- | --- |
| temp | 3.46 | [2.48, 5.07] | 1.86 | 0.29 | [0.20, 0.40] |
| HUM | 2.88 | [2.11, 4.20] | 1.706 | 0.35 | [0.24, 0.47] |
| DE | 1.49 | [1.21, 2.14] | 1.22 | 0.67 | [0.47, 0.83] |
| DA | 1.60 | [1.28, 2.30] | 1.27 | 0.62 | [0.44, 0.78] |
| Shelter | 1.39 | [1.15, 2.02] | 1.18 | 0.72 | [0.50, 0.87] |
| resource | 3.51 | [2.52, 5.15] | 1.87 | 0.29 | [0.19, 0.40] |
| P_resource | 3.31 | [2.79, 4.84] | 1.82 | 0.30 | [0.21, 0.42] |
| Subs_H | 1.63 | [1.30, 2.34] | 1.28 | 0.61 | [0.43, 0.77] |

Table S6. Best models for invertebrate richness in the quadrats. Model selection according to the corrected Akaike criterion for small sampling size. We only listed the models with delta < 2. AICc. Corrected Akaike information criterion for small sample size; delta AICc. (AIC of the model - AIC of the best model); Akaike weight; Marginal R². variance explained by the fixed effects alone; Conditional R². variance explained by both the fixed and random effects together.

| cond(DA) | cond(DE) | cond(HUM) | cond(p_resource) | cond(resource) | cond(shelter) | cond(subs_H) | cond(temp) | df | AICc | delta | weight | Marginal R² | Conditional R² |
| --- | --- | --- | --- | --- | --- | --- | --- | --- | --- | --- | --- | --- | --- |
|  |  | -0.162 |  | 0.278 | 0.271 |  |  | 5.000 | 545.146 | 0.000 | 0.141 | 0.156 | 0.355 |
| -0.110 |  | -0.190 |  | 0.293 | 0.267 |  |  | 6.000 | 545.572 | 0.426 | 0.114 | 0.176 | 0.367 |
|  | -0.092 | -0.123 |  | 0.274 | 0.261 |  |  | 6.000 | 545.870 | 0.724 | 0.098 | 0.160 | 0.349 |
|  |  | -0.158 |  | 0.285 | 0.238 | 0.096 |  | 6.000 | 545.886 | 0.740 | 0.097 | 0.171 | 0.358 |
| -0.117 | -0.099 | -0.151 |  | 0.289 | 0.256 |  |  | 7.000 | 546.074 | 0.928 | 0.089 | 0.183 | 0.362 |
|  |  | -0.181 | + | 0.218 | 0.260 |  |  | 6.000 | 546.199 | 1.052 | 0.083 | 0.164 | 0.352 |
|  | -0.139 |  |  | 0.273 | 0.280 |  |  | 5.000 | 546.251 | 1.105 | 0.081 | 0.151 | 0.349 |
|  | -0.104 | -0.139 | + | 0.202 | 0.247 |  |  | 7.000 | 546.522 | 1.376 | 0.071 | 0.170 | 0.345 |
| -0.102 |  | -0.206 | + | 0.236 | 0.256 |  |  | 7.000 | 546.863 | 1.716 | 0.060 | 0.180 | 0.363 |
| -0.091 |  | -0.183 |  | 0.296 | 0.242 | 0.074 |  | 7.000 | 546.953 | 1.807 | 0.057 | 0.184 | 0.368 |
| -0.109 | -0.110 | -0.164 | + | 0.221 | 0.243 |  |  | 8.000 | 547.003 | 1.857 | 0.056 | 0.188 | 0.356 |
|  | -0.076 | -0.126 |  | 0.281 | 0.236 | 0.079 |  | 7.000 | 547.113 | 1.967 | 0.053 | 0.172 | 0.353 |

Figure S9. Results of DHARMa residual for the Poisson GLMM of invertebrate richness across quadrats. Left panel creates a qq-plot to detect overall deviations from the expected distribution. by default with added tests for correct distribution (KS test). dispersion and outliers (Hartig. 2024; https://cran.r-project.org/web/packages/DHARMa/vignettes/DHARMa.html).

Figure S10. Result of DHARMa zero-inflation test for the Poisson GLMM of invertebrate richness across quadrats.

Table S7. Variance inflation factors (VIFs) for the Poisson GLMM of invertebrate richness across quadrats.

| Term | VIF | VIF 95% CI | Increased SE | Tolerance | Tolerance 95% CI |
| --- | --- | --- | --- | --- | --- |
| temp | 2.09 | [1.72, 2.64] | 1.44 | 0.48 | [0.38, 0.58] |
| HUM | 1.94 | [1.61, 4.45] | 1.39 | 0.51 | [0.41, 0.62] |
| DE | 1.38 | [1.20, 1.73] | 1.17 | 0.73 | [0.58, 0.84] |
| DA | 1.37 | [1.19, 1.72] | 1.17 | 0.73 | [0.58, 0.84] |
| Shelter | 1.46 | [1.26, 1.83] | 1.21 | 0.68 | [0.55, 0.80] |
| resource | 1.21 | [2.52, 5.15] | 1.49 | 0.45 | [0.36, 0.55] |
| P_resource | 2.06 | [1.70, 2.61] | 1.44 | 0.48 | [0.38, 0.59] |
| Subs_H | 1.44 | [1.24, 1.81] | 1.20 | 0.69 | [0.55, 0.81] |

Figure S11. Rarefaction and extrapolation of species richness (A and C) and sample coverage (B and D) using abundance data with 95% confidence intervals (shaded areas).

Figure S12. Rarefaction and extrapolation of species richness (A and C) and sample coverage (B and D) using incidence data with 95% confidence intervals (shaded areas).

**References**

Berbert-Born M, Karmann I (2002) Lapa dos Brejões-Vereda Romão Gramacho, Chapada Diamantina, BA. In: Sítios geológicos e paleontológicos do Brasil, 2. Available in: https://www.sigep.eco.br/sitio016/sitio016.pdf

Deharveng L, Bedos A, Pipan T, Culver DC (2024) Global subterranean biodiversity: a unique pattern. Diversity 16(3):157. <https://doi.org/10.3390/d16030157>

Furtado-Oliveira L, Ferreira RL, Fernández JIR, Souza-Silva M (2022) Recreational caving impacts of visitors in a high-altitude cave in Bolivian Andes: main effects on microhabitat structure and faunal distribution. Int J Speleol 51(2):2. <https://doi.org/10.5038/1827806X.51.2.2418>

Hsieh TC, Ma KH and Chao A (2016) iNEXT: an R package for rarefaction and extrapolation of species diversity (Hill numbers). Methods Ecol Evol 7: 1451-1456. https://doi.org/10.1111/2041-210X.12613

Sket B (2008) Can we agree on an ecological classification of subterranean animals? J Nat Hist 42(21–22):1549–1563. <https://doi.org/10.1080/00222930801995762>

Souza-Silva M, Cerqueira RFV, Pellegrini TG, Ferreira RL (2021) Habitat selection of cave-restricted fauna in a new hotspot of subterranean biodiversity in Neotropics. Biodivers Conserv 30(14):4223–4250. <https://doi.org/10.1007/s10531-021-02302->8

Souza-Silva M, Lourenço-Guimarães LC, Ferreira RL (2025) Subterranean biodiversity in the largest limestone cave of the Amazon: factors shaping terrestrial invertebrate diversity across sampling scales. J Insect Biodivers 71(1):15–33. DOI:10.12976/jib/2025.71.1.2

Zampaulo RDA, Souza-Silva M, Ferreira RL (2025) Subterranean biodiversity on the brink: urgent framework for conserving the densest cave region in South America. Animals 15(19):2899. <https://doi.org/10.3390/ani15192899>
